# Into the Dynamics of a Supramolecular Polymer at Submolecular Resolution

**DOI:** 10.1038/s41467-017-00189-0

**Published:** 2017-07-27

**Authors:** Davide Bochicchio, Matteo Salvalaglio, Giovanni M. Pavan

**Affiliations:** 1 Department of Innovative Technologies, University of Applied Sciences and Arts of Southern Switzerland, Galleria 2, Via Cantonale 2c, CH-6928 Manno, Switzerland; 2 Thomas Young Centre and Department of Chemical Engineering, University College London, London, WC1E 7JE UK

## Abstract

To rationally design supramolecular polymers capable of self-healing or reconfiguring their structure in a dynamically controlled way, it is imperative to gain access into the intrinsic dynamics of the supramolecular polymer (dynamic exchange of monomers) while maintaining a high-resolution description of the monomer structure. But this is prohibitively difficult at experimental level. Here we show atomistic, coarse-grained modelling combined with advanced simulation approaches to characterize the molecular mechanisms and relative kinetics of monomer exchange in structural variants of a synthetic supramolecular polymer in different conditions. We can capture differences in supramolecular dynamics consistent with the experimental observations, revealing that monomer exchange in and out the fibres originates from the defects present in their supramolecular structure. At the same time, the submolecular resolution of this approach offers a molecular-level insight into the dynamics of these bioinspired materials, and a flexible tool to obtain structure-dynamics relationships for a variety of polymeric assemblies.

## Abstract (web summary)

Accessing the dynamics of soft self-assembled materials at high resolution is very difficult. Here the authors show atomistic and coarse-grained modelling combined with enhanced sampling to characterize the molecular mechanisms and kinetics of monomer exchange in synthetic supramolecular polymers.

## Introduction

Synthetic supramolecular polymers, where monomers are interconnected through noncovalent interactions, possess dynamic and responsive properties reminiscent of many natural materials, which make them extremely interesting for various applications^1–7^. Many self-assembling motifs generating one-dimensional fibres in various media have been reported^8–18^. While a first interesting point is how the information stored into the monomers is transmitted to the structure of the fibre, the intrinsic dynamics of supramolecular polymers is perhaps even more interesting^19–21^, but far more elusive.

**Fig. 1:**
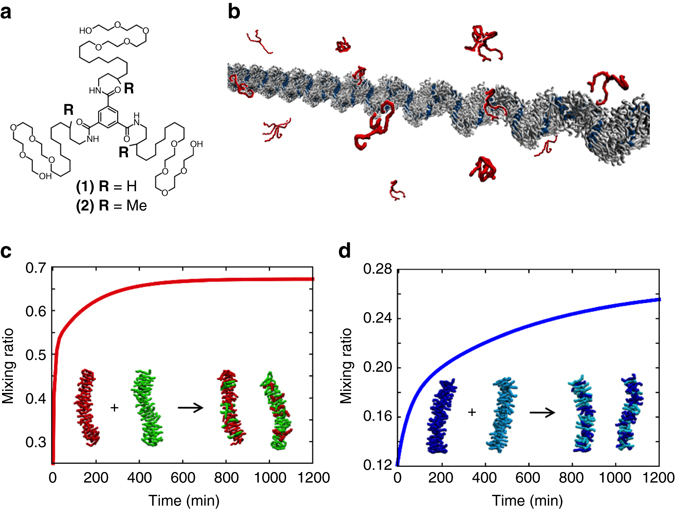
Previous experiments on monomer exchange in dynamic water-soluble BTA supramolecular polymers. **a** Chemical structures of water-soluble BTA monomers (**1**) and (**2**). **b** Monomer exchange in and out these supramolecular polymers (equilibrium between the assembled monomers and the monomers in solution) occurs uniformly along the fibre length (previous STORM experiments)^22^. **c**, **d** Fitting curves for the exchange kinetics in supramolecular fibres (**1**) and (**2**) (from FRET mixing essay)^23, 25^. All terms in these multi-exponential exchange curves were found ~1 order of magnitude slower in fibre (**2**) compared to (**1**), where fibre (**1**) reached mixing equilibration within ~2 h **c** while (**2**) did not plateau even after ~20 h **d** ^25^

These directional assemblies continuously exchange monomers with the surrounding in a dynamic way (Fig. 1)^19–21^, while the rate of this exchange is key for bioinspired properties such as the ability of the fibre to self-heal or dynamically reconfigure its structure. The origin of such innate dynamics is deeply encoded into the structure of the monomers, which poses the intriguing/ambitious goal of rationally designing the monomers to control the dynamics of the supramolecular polymer.

Perfecting a method/technique to assess the dynamic exchange of monomers in and out supramolecular polymers is a first challenging task from an experimental point of view, especially in aqueous solution. For example, STochastic Optical Reconstruction Microscopy (STORM)^22^ and Förster Resonance Energy Transfer (FRET)^23^ have been recently used to study the dynamics of water-soluble 1,3,5-benzenetricarboxamides (BTA) supramolecular polymers (Fig. 1a), where the BTA monomers directionally self-assemble via threefold hydrogen bonding and stacking of cores^9^. The BTA motif can be readily modified to provide solubility in various environments^9, 24^, which makes it ideally suited for fundamental studies on supramolecular polymers. Unlike microtubules, where polymerization/depolymerization occurs at the tips, recent STORM experiments (~20 nm resolution) showed uniform exchange along water-soluble BTA fibres^22^. FRET mixing essay also allowed to monitor the equilibration in time of monomer exchange between fibres in solution^23^. Comparing water-soluble fibres of BTA monomers (**1**) with amphiphilic side arms (C_12_-PEG_4_-OH) to a BTA analogue (**2**)^24^, where a subtle modification – a stereogenic methyl centre—was added into the side chains of the monomers (Fig. 1a), demonstrated that (**2**) fibres exchange much slower than (**1**) fibres^25^. The exchange kinetics was found to fit well with a bi-exponential process (at least two exponentials were needed for the fitting), where both slow and fast timescales differ by ~1 order of magnitude between the two fibres (Figs. 1c, d)^25^. The physical origin of this multi-exponential behaviour remained unclear. However, these experimental results suggested that monomer exchange in these systems is likely not a single-step phenomenon, but rather a complex process involving multiple steps with faster/slower kinetics; and that both fibres seem to exchange in similar fashion, while all main exchange steps are (comparably) slower in fibre (**2**) compared to fibre (**1**)^25^. Yet, despite these experimental advances, a detailed molecular-level understanding of monomer exchange in these supramolecular polymers remained unattainable due to the limited resolution achievable in the kinetic experiments.

In the absence of molecular-level experimental details, all-atom (AA) and coarse-grained (CG) molecular dynamics (MD) simulations were recently proven extremely useful to obtain high-resolution insights into both structure and thermodynamics of supramolecular polymers that could not be obtained by the experiments^25–31^. However, within the limited timescales effectively accessible in such MD simulations, monomer exchange is a rare event, which hindered the study of the dynamics of these supramolecular polymers. Inspired by recent computational developments that allow simulating rare events occurring on timescales largely exceeding those accessible in classical MD simulations^32–36^, here we used well-tempered metadynamics (WT-MetaD)^37^ simulations to study monomer exchange in BTA supramolecular polymers in various solvents at submolecular resolution. With this approach, we can provide molecular-level description of the mechanism, pathway and kinetics of monomer exchange that have never been achieved so far.

## Results

### Mechanism of exchange in organic solvent and in gas phase

**Fig. 2:**
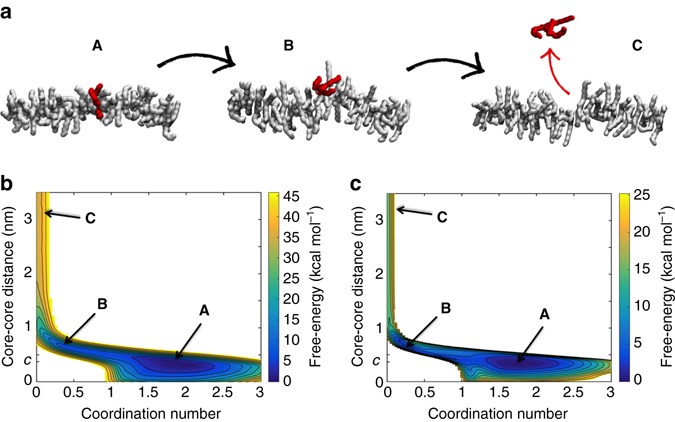
Mechanism of monomer exchange in BTA-C_6_ fibres in the gas phase or in organic solvent. **a** A stepwise exchange process: WT-MetaD activated exchange of a monomer (*red*) from the centre of BTA-C_6_ 24-mers (*grey*) demonstrates that a defect/breakage must be first created along the stack (**A**–**B** transition) from which the monomer can jump out from the fibre (**B**–**C** transition). **b**, **c** Free-energy surfaces (FESs) for monomer exchange in the gas phase **b** and organic solvent (**c**: pentane, C_5_) as a function of the minimum distance between the activated core and the other ones in the 24-mers, and of core-core coordination (see Supplementary Methods for details). *Blue* and *yellow* colours in the FES identify energetically favourable or unfavourable regions respectively, revealing the exchange pathway. The same stepwise exchange mechanism is observed independent of the solvent—going from **A** to **C** requires **B** (creation of a defect)

Focusing on BTA supramolecular polymers as a case study, we availed of the high-resolution and flexibility of atomistic and molecular models to attain a detailed understanding of monomer exchange. We started from simple supramolecular fibres of BTA monomers with reduced side chains (BTA-C_6_) in the gas phase. We also studied the same system in organic solvent (pentane, C_5_), where the BTA-C_6_ monomers are soluble. In these conditions this system is known to form ordered/extended stacks^9, 25, 26^. Atomistic models of pre-stacked BTA-C_6_ 24-mers (Fig. 2) were preliminarily equilibrated via AA-MD simulations as immerged in explicit C_5_ molecules as well as in the gas phase (Methods section). These two simulated systems are extremely useful to our study, as these allow investigating monomer exchange in intrinsically ordered, relatively simple supramolecular polymers (less structurally complex, for example, than water-soluble BTA fibres: see below).

Various computational approaches have been used to study rare events in complex molecular systems^32–36, 38–43^. Well suited for our case, the group of Parrinello recently used WT-MetaD simulations to study, for example, drug unbinding from protein binding pockets^33^, or the condensation of Argon droplets from supersaturated vapour^34^. We adapted this approach to study monomer exchange in BTA supramolecular polymers.

We activated monomer exchange from the central region of BTA-C_6_ 24-mers via WT-MetaD simulations (Methods section and Supplementary Methods). During the WT-MetaD runs, the activated monomer (Fig. 2a: red) was seen to leave the oligomer, exchanging with the surrounding and being subsequently reincorporated into the stack. From the WT-MetaD simulations we obtained the free-energy surfaces (FES) for the monomer exchange event in the different environments (Figs. 2b, c: FES in the gas phase and C_5_).

Our WT-MetaD simulations reveal that monomer exchange from these stacks is a stepwise process. Darkest colours in the FES indicate minimum energy configurations for the systems, identifying the intermediate states and overall exchange pathway. In all cases, the global minimum energy (most favourable) configuration for the system is found at stacking distance (*c* ~3.5 Å) and monomer coordination ~2, corresponding to the monomer as perfectly stacked in the 24-mer (**A**). In their exchange pathway, BTA-C_6_ monomers do not diffuse directly from the 24-mers to the solvent (**A–C**). A local breakage/defect (**B**) is first created along the stack (monomer attached by one end: low intercore distance and reduced monomer coordination), from which the monomer can leave the oligomer (**C**). The **B–C** transition is characterized by a free-energy penalty that depends on monomer solubility in the various environments (~15 kcal mol^−1^ in C_5_, while in the gas phase this increases to ~30 kcal mol^−1^). The **A-B** transition is found easier in organic solvent than in the gas phase, where the ∆*G* required for the creation of one defect along the stack, respectively, increases from a few kcal mol^−1^ to ~10–15 kcal mol^−1^. This is consistent with the intrinsically ordered configuration of the stack in the gas phase and with the increased mobility of the BTA-C_6_ monomers in organic solvent. While here we are primarily interested in water-soluble supramolecular polymers, simulating these simpler systems is very useful to obtain a first molecular-level insight into the mechanism of monomer exchange in these supramolecular fibres, highlighting the importance of defects in the process.

### Exchange hot spots in water-soluble supramolecular polymers

Dynamic supramolecular polymers that are soluble in water are extremely interesting for many applications (biomaterials, tissue engineering, etc.)^1, 3–7, 15–18^. However, while in the gas phase and organic solvents the study of monomer exchange benefits from the simplified structure of the monomers and the intrinsic order in the stacks, complexity greatly increases in water due to the size/flexibility of water-soluble monomers (Fig. 1a) and the importance of hydrophobic effects.

**Fig. 3:**
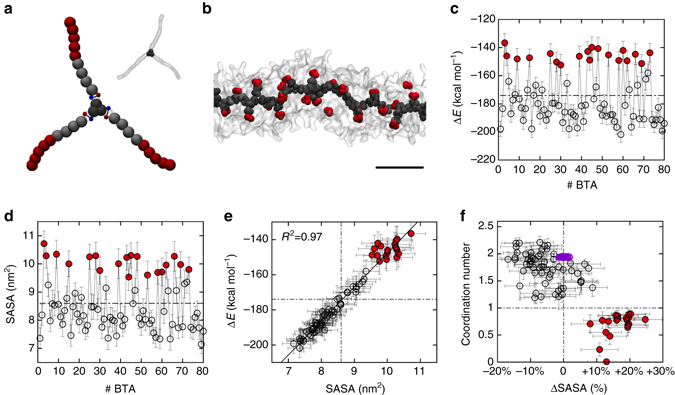
Exchange hot spots in water-soluble BTA supramolecular polymers. **a** CG model for BTA monomer (**1**):^31^ aromatic core in *dark grey*, ±*q* charges (explicit H-bonding treatment) in the amides in *red* and *blue*, dodecyl spacers in *light grey* and PEG in *red*. **b** Equilibrated configuration of fibre (**1**) in water obtained via CG-MD simulation – BTA cores are shown in *dark grey* (and *red*), the amphiphilic branches of the BTA water-soluble monomers are shown in *transparent grey*, water not shown for clarity (scale bar: 3 nm). **c** Interaction energy (∆*E*) of each monomer with the rest of fibre (**1**). **d** Solvent accessible surface area (SASA) for each monomer in equilibrated CG-fibre (**1**). Hot spot monomers (*red*) have ∆*E* and SASA, respectively, less favourable and larger than the average (*dash dotted lines*). **e** Inverse linear relationship between the strength of incorporation (∆*E*) and monomer exposure to the solvent (SASA) (Supplementary Figs 1, 2: same data for CG-fibre (**2**) and AA-fibre (**1**)). **f** Monomer coordination vs. SASA (percentage deviation from the average monomer SASA). Black points refer to fibre (**1**): monomers with coordination ≤ 1 and SASA greater than the average correspond to hot spots (*red*). *Purple*: same data for CG BTA-C_6_ fibre in organic solvent – no defects (*hot spots*) are present along these ordered stacks (see also Supplementary Fig. 3). Error bars in the plots represent s.e.m

Recently, we developed all-atom (AA) and coarse-grained (CG) models of supramolecular polymers (**1**) and (**2**) consistent with the experimental evidences available for these systems (Fig. 3a: GC monomer (**1**))^25, 30, 31^. Our simulations demonstrated that these stacks are not perfect in water, where fibre folding due to hydrophobic effects produces multiple defects/breakages along the supramolecular polymers (Fig. 3b)^25, 30, 31^. Analysis of the incorporation energy (∆*E*) and solvent accessible surface area (SASA) of each monomer in the equilibrated AA and CG models of these BTA stacks reveals that, in correspondence of these defected points, monomers are more weakly incorporated into the fibre and more exposed to the solvent than the average (Figs. 3c, d: red monomers with ∆*E* less favourable than ∆*E*_avg_ and SASA greater than SASA_avg_). Plotting ∆*E* against SASA for each monomer produces a nearly perfect linear trend (Fig. 3e: *R* ^2^ > 0.97 for CG-fibre (**1**); Supplementary Fig. 1: same plots for CG-fibre (**2**)). Similar trend and defects in fibre (**1**) are evidenced also at AA-level (Supplementary Fig. 2: AA-fibre (**1**))^25^. While AA models are clearly more static than CG ones (limited in terms of sampling, length and timescales that can be effectively explored), this demonstrates that even at such a high-resolution core stacking within BTA fibre (**1**) is far from being perfect in water.

It is interesting to relate the coordination number of each BTA core to the SASA (percentage deviation from the average) for the individual monomers in the fibre (Fig. 3f). This allows to unambiguously identify defects in the fibre—monomers with coordination ≤ 1 and SASA greater than the average (Fig. 3f: red)—and to compare between different fibres (different size monomers). For example, for a fair comparison with the ordered stacks of Fig. 2, we also created a CG model for a BTA-C_6_ fibre in organic solvent (Methods section). It is interesting to note that the same analyses from the CG-MD simulation of this system (Supplementary Fig. 3) do not evidence the spontaneous formation of any persistent defect/breakage in the timescales accessible with our model (microseconds). Unlike fibre (**1**), CG-fibre BTA-C_6_ has all monomers with coordination ~2 and SASA well consistent with the average value, which highlights the nearly perfect nature of these stacks (Fig. 3f: purple).

Thus, while in intrinsically ordered fibres (BTA-C_6_ in gas phase and organic solvents) we observe that monomer exchange can proceed provided that a defect is first created along the stack, water-soluble BTA fibres intrinsically possess a number of hot spots where monomer exchange is more likely to occur.

### Monomer exchange in a water-soluble BTA supramolecular fibre

**Fig. 4:**
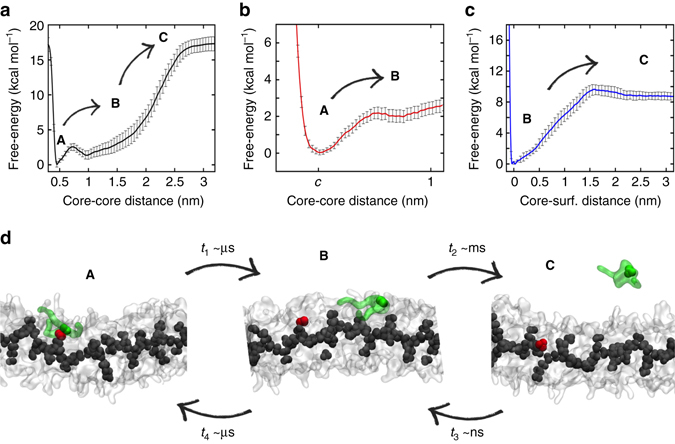
Monomer exchange in water-soluble fibre (**1**) modelled via WT-MetaD simulations. **a** Free-energy profile of monomer exchange with water as a function of the minimum distance between the activated core and the other cores in fibre (**1**). Starting from a configuration where the activated monomer (*green* in the snapshots) is stacked onto a hot spot (*red*) on the fibre surface (**A**), the monomer does not diffuse directly in water **C**, but tends to diffuse onto the surface of fibre (**1**) (Step 1: **A** to **B**) before jumping in the solvent (Step 2: **B** to **C**). **b** Quantitative FES for Step 1 as a function of the minimum core-core distance. **c** Quantitative FES for Step 2 as a function the distance between the activated monomer and the fibre surface. Error bars in the FESs represent s.e.m. **d** CG-WT-MetaD snapshots representing the **A**–**B** and **B**–**C** transitions and the relative timescales (*t*_1_ and *t*_2_) (water not shown for clarity). The spontaneous incorporation of a dissolved monomer into fibre (**1**) follows the reverse stepwise process (unbiased CG-MD): the monomer (*t*_3_) collapses onto fibre (**1**) (Step 3) and diffuses on the surface, moving from one hot spot to another (Step 4) with residence time of *t*_4_ (equal to *t*_1_ from WT-MetaD). Exchanging a monomer from the fibre interior (Steps 0–2) is way more infrequent than exchanging monomers present on the fibre surface (see Supplementary Fig. 5a for all computed timescales)

We started from this evidence to investigate in detail the exchange of monomers in and out water-soluble BTA supramolecular polymers (**1**) and (**2**). Due to the excessive complexity of these systems at atomistic-level, we relied on our CG models for fibres (**1**) and (**2**), simpler than AA ones but comparably accurate in the treatment of these supramolecular polymers (Methods section)^31^. Starting from system (**1**), we chose one monomer (Fig. 4d: green) corresponding to a surface hot spot in equilibrated CG-fibre (**1**) (Fig. 3: red). WT-MetaD simulations activating monomer exchange from the hot spot revealed that monomer exchange with water is not a single-step process (**A–C**). After leaving the hot spot, monomers tend to diffuse onto the fibre surface (Step: 1, **A–B** transition) before jumping into water (Step: 2, **B–C**). Figure 4a reports the free-energy profile for monomer exchange as a function of the minimum distance between the activated monomer (Fig. 4d: green) and its closest neighbour in the hot spot (red). Such profile is characterized by two minima, a first one (**A**) at stacking distance *c* (global minimum), and a second one at greater distance (**B**). The activated exchange of other monomers from different hot spots showed the same stepwise exchange pathway.

**Fig. 5:**
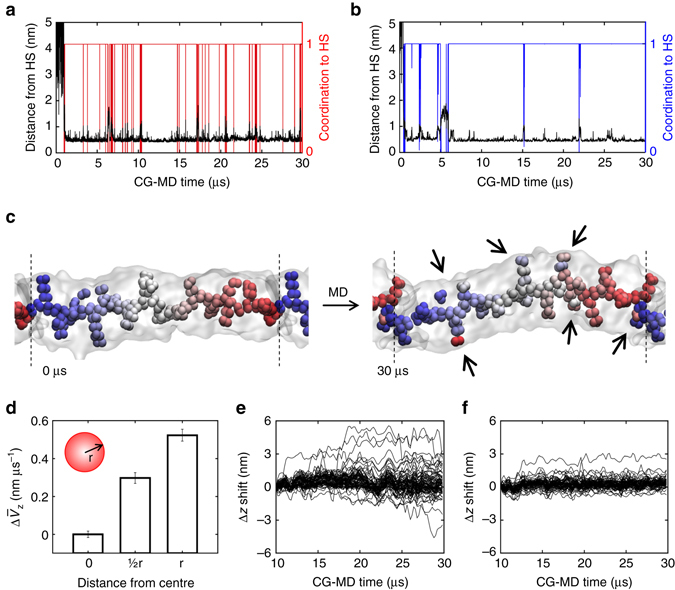
Internal dynamics of water-soluble BTA supramolecular polymers. **a**, **b** Spontaneous incorporation (CG-MD) of a dissolved monomer into fibres (**1**) and (**2**) and monomer diffusion between the surface hot spots. *Black*: minimum distance between the initially dissolved monomer (core) and the other cores in the fibres. *Red*, *blue*: monomer coordination (core) to the closest surface hot spot (HS) in fibres (**1**) **a** and (**2**) **b**, respectively,. After ~1 µs the monomer collapses on the fibre surface and stacks onto a hot spot (coordination rising to 1). Every time that HS coordination drops to 0 the monomer leaves the hot spot and stacks onto another one. **c** Initial and final (30 µs) snapshots (CG-MD) of fibre (**1**): BTA cores coloured based on their initial *z*-displacement, PEG and water beads not shown for clarity. Monomer shifting along the main fibre axis (*z*) occurs on the surface rather than in the core (*black* arrows: colour mixing/mismatches). **d** Average monomer velocity along *z* in fibre (**1**) ($\Delta {\overline v _z}$, calculated respect to the fibre core) as a function of the distance from the fibre centre (error bars: s.e.m.). **e**, **f** Monomer shifting along the fibres (∆*z*): some monomers move back and forth along fibre (**1**) **e**, while fibre (**2**) is more static **f**

Quantitative FESs for the individual **A–B** and **B–C** transitions (Fig. 4b, c) were calculated with different approaches, proving the reliability of these results (Methods section and Supplementary Fig. 4). The **A–B** FES (Fig. 4b) shows that to leave the hot spot (**A**) and diffuse onto the surface of fibre (**1**) (**B**) monomers have to cross a small free-energy barrier (~2 kcal mol^−1^), corresponding to a relatively frequent event at room temperature. In fact, we can routinely observe this transition even during unbiased CG-MD simulations (see Fig. 5b). On the other hand, monomer jumping from the surface into water (**B–C** transition) requires more energy (Fig. 4c: ~10 kcal mol^−1^) and is never observed during unbiased simulations.

Using multiple infrequent WT-MetaD simulations we calculated characteristic timescales for the key steps in monomer exchange^32, 35^—Step 1 (*t*_1_): **A–B** transition; Step 2 (*t*_2_): **B–C** transition (Methods section). Shown in Fig. 4d, in fibre (**1**) *t*_1_ is found in the timescale of µs (~10^−6^ s) and *t*_2_ in that of ms (~10^−3^ s). While these collected timescales pertain to CG models and have qualitative/comparative value, this finding clearly demonstrates that in fibre (**1**) Step 1 is orders of magnitude faster than Step 2. This is consistent with recent hypotheses based on STORM experiments, suggesting that the internal dynamics of this fibre is faster than monomer exchange with water^22^.

Unbiased CG-MD simulations modelling the spontaneous incorporation of a dissolved monomer into equilibrated fibre (**1**) clearly demonstrate that this process follows the reverse stepwise pathway (Fig. 4d). The monomer collapses very rapidly (*t*_3_) onto the surface of the supramolecular polymer (**C–B**), and then diffuses on the latter until reaching an accessible hot spot (**B–A**). Multiple CG-MD simulations where the monomers are incorporated into the fibre from different positions demonstrate the reproducibility of these observations. On longer timescales, the reincorporated monomer can leave state (**A**) moving from one hot spot to another onto the fibre surface, while the characteristic residence time in state (**A**) is of ~µs (*t*_4_) (Fig. 5a: every time that red coordination to hot spots drops to 0, see also below). The FES and characteristic timescale (*t*_4_) for the **A–B** transition calculated from these unbiased CG-MD simulations (Methods section) are found identical to those from WT-MetaD (*t*_1_), confirming the reliability of our approach.

**Fig. 6:**
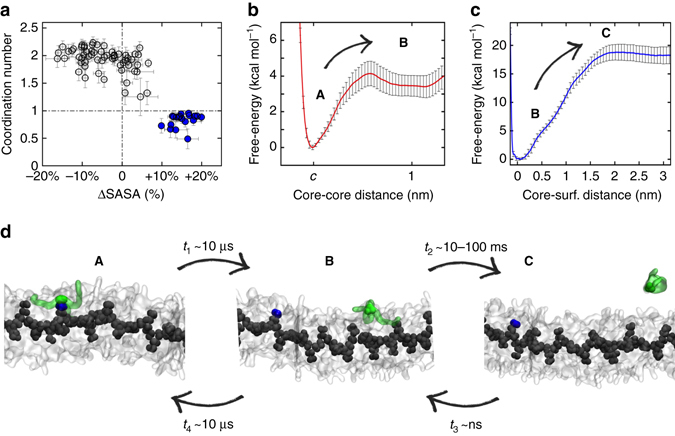
Monomer exchange in water-soluble fibre (**2**) modelled via WT-MetaD simulations. **a** Monomer coordination vs. monomer SASA (percentage deviation from the average): also fibre (**2**) possesses defects (*blue*: hot spot monomers with coordination ≤ 1) similar to those of fibre (**1**). Monomer exchange between the surface hot spots and water in fibre (**2**) follows the same stepwise mechanism than in fibre (**1**): **A**–**C** = **A**–**B** (Step 1)** + B**–**C** (Step 2). **b**, **c** FESs for Step 1 **b** and Step 2 **c**. Error bars in the FESs represent s.e.m. **d** Snapshots representing the **A**–**B** and **B**–**C** transitions in fibre (**2**) and the relative transition timescales (*t*_1_ and *t*_2_) collected from the CG-WT-MetaD simulations (water not shown for clarity). The spontaneous incorporation of a dissolved monomer (*green*) into fibre (**2**) (unbiased CG-MD) follows the reverse stepwise process. Also in fibre (**2**), exchanging a monomer from the fibre interior (Steps 0–2) is more unfavourable/slower than exchanging monomers present on the surface (Supplementary Fig. 5a)

We also activated via WT-MetaD monomer exchange from the interior of the fibre (Fig. 3: monomers with coordination ~2, ∆*E* more favourable and SASA lower than the average). Consistent with what seen in the gas phase and organic solvents (Fig. 2), in this case the system first needs to create a new hot spot on the fibre surface (Step 0) from which monomer exchange can then proceed via Steps 1 and 2 (Supplementary Movie 1). However, we could observe that Step 0 is very slow (*t*_0_ ~0.1–1 s) compared to the latters (Fig. 6d: *t*_1_ and *t*_2_), which makes exchanging monomers from the interior of the fibre (Steps 0–2) an unfavourable, less probable event than exchanging monomers already present on the fibre surface, either as adsorbed (Step 2) or stacked onto the hot spots (Steps 1–2) (see Supplementary Fig. 5a for all computed timescales). The same can be said for the exchange of groups/aggregates of monomers. For example, WT-MetaD simulations activating the exchange of dimers between the surface of fibre (**1**) and water showed that, while possible, this event is extremely unfavourable and infrequent compared to the exchange of monomers (Methods section and Supplementary Fig. 6: *t*_2_(**1**)_dimer_ >> *t*_2_(**1**)_monomer_).

These findings indicate that monomer exchange preferentially originates from the numerous hot spots identified in our models (defects) present all along the fibre surface. By bridging the gap between structure and dynamics, our approach provides molecular-level insight consistent with the uniform exchange along fibre (**1**) seen by STORM, explicable only under the conditions that core stacking is not perfect and defected points prone to monomer exchange (our hot spots) are present all along these supramolecular polymers^22^.

### Effect of molecular structure on monomer exchange dynamics

We studied monomer exchange in supramolecular polymer (**2**), where (**2**) monomers differ from (**1**) only by a methyl group in the side chains (Fig. 1a). Although at this level of accuracy this is a relatively subtle change, our CG models already demonstrated the ability to capture differences between fibres (**1**) and (**2**) consistent with the experimental trends^31^. Analysis of order/disorder demonstrates that CG-fibre (**2**) possesses defects (hot spots) along the stack similar to those of fibre (**1**), although in this case these are found more static (Fig. 6a, blue). Although (**2**) monomers are larger than (**1**), the average monomer SASA is found smaller in fibre (**2**) than in (**1**) (Supplementary Fig. 1d vs. Fig. 3e): (**2**) monomers are more hydrophobic and tightly compacted in fibre (**2**).

Shown in Fig. 6b–d, all monomer exchange data collected for supramolecular polymer (**2**) reveal the same stepwise mechanism seen for fibre (**1**). However, in this case Steps 0–2 are all found ~1–2 orders of magnitude slower than in fibre (**1**) (Fig. 6d and Supplementary Fig. 5a). This fits well with the difference of ~1 order of magnitude seen in all terms of the previously reported FRET exchange curves for the two fibres^25^, suggesting that the key exchange steps are all comparably slower in fibre (**2**) than in (**1**).

The spontaneous incorporation of dissolved monomers into supramolecular polymer (**2**) via unbiased CG-MD also shows the same reverse stepwise mechanism seen for fibre (**1**). However, monomer unbinding and diffusion between hot spots can be only sparsely observed during 30 µs of CG-MD of fibre (**2**) (Fig. 5b: blue). Coherent with *t*_1_ from WT-MetaD, in this case the residence time of monomer (**2**) in the hot spots is found ~10 times larger than in system (**1**) (*t*_4_ ~10 µs on average). Reliable timescales for the **A–B** transition could be obtained also from the AA models of these fibres via WT-MetaD (Methods section). Also in this case, *t*_1_ was found ~1 order of magnitude slower in fibre (**2**) compared to (**1**) (Supplementary Fig. 5b), in line with the difference seen in our CG models and with the experimental trends^25^.

### Internal dynamics of water-soluble BTA supramolecular fibres

Monomer mobility on the surface of these supramolecular polymers is extremely interesting (Supplementary Movie 2). Monomers are seen to diffuse along the fibres even during 30 µs of unbiased CG-MD simulation (Fig. 5c: mixing of coloured monomers in fibre (**1**) identified by black arrows). However, the residence time of monomers onto the hot spots, and overall fibre dynamics, depends on the monomer structure, as well demonstrated by the hot spot coordination data (exchange between the hot spots) extracted from the unbiased CG-MD simulation for fibres (**1**) and (**2**) (Fig. 5a, b). Interestingly, we can observe that the largest monomer movements occur in proximity of the surface, while the internal core of the fibres appears as more static (Fig. 5c: *z*-displacement of central coloured cores is substantially preserved during CG-MD). Monitoring the shifting of the individual (80) monomers along the main axis of the fibre models (∆*z* shift) reveals that these supramolecular polymers possess different levels of internal dynamics. The average monomer drift velocity ($\Delta {\overline v _z}$) as a function of the distance from the fibre centre demonstrates that surface monomers move faster than those in the core (Fig. 5d). For example, in fibre (**1**) some monomers are seen to spontaneously move back and forth along fibre (**1**) during CG-MD (Fig. 5e: maximum ∆*z* shifts of ~ + 5 nm and ~−3 nm over a total fibre length of ~13 nm), while fibre (**2**) is less dynamic in general (Fig. 5f). The dynamic diversity of these supramolecular polymers is reminiscent of many materials in Nature, where the constitutive building blocks exposed to the external environment are more dynamic than those in the interior.

### Key factors for supramolecular dynamics

Comparing the dynamics of fibres (**1**) and (**2**) we can observe that increasing the hydrophobicity of the monomers ((**2**) vs. (**1**)) make the fibre more static in general, reducing both the rate of monomer exchange in and out (exchange with water) and within the fibre (internal dynamics). Interesting question arises: Can we control one process over the other one ? How far can we push our comprehension of the factors that control the dynamics of these fibres?

**Fig. 7:**
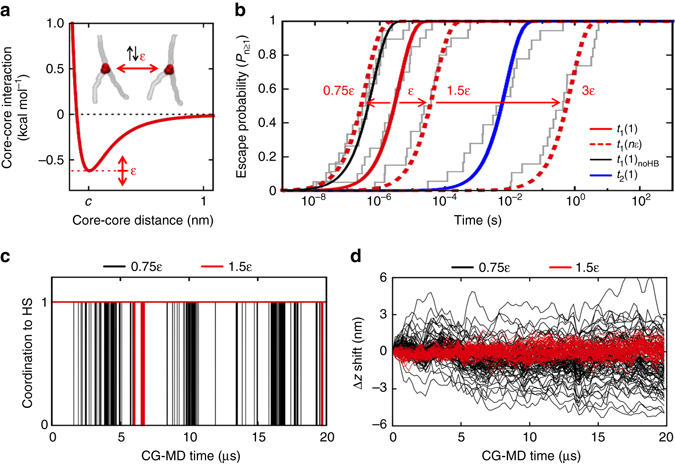
Key factors controlling the dynamics of water-soluble BTA supramolecular polymers. **a** Toy model: the core-core interaction (ε) between the monomers in CG-fibre (**1**) was systematically strengthened/weakened without changing any other solute-solute or solute-solvent interaction in the model. **b** Transition time distributions for the monomer exchange Step 1 obtained via multiple infrequent WT-MetaD simulations (each grey segment in the distributions is one WT-MetaD run). Poisson distribution fitting curves – solid red for the original (ε), dotted red for 1.5ε, 3ε and 0.75ε – providing the characteristic timescales for Step 1 (*t*_1_) as a function of increasing/decreasing the ε. Acceleration of Step 1 analogous to that seen at 0.75ε, is also obtained deleting the H-bonding between the monomers ((**1**)_noHB_: black). Step 2 (*t*_2_), invariant in all these cases, is reported in blue for comparison. **c** Spontaneous monomer diffusion (CG-MD) between the surface hot spots: monomer coordination in 1.5ε (red) and 0.75ε (black) systems. **d** Monomer shifting (∆*z*) along 1.5ε (red) and 0.75ε (black) fibres (Supplementary Fig. 7: same data for (**1**)_noHB_)

We used CG-fibre (**1**) as a basis for the development of a toy model in which the interaction strength between the BTA cores (ε) was systematically increased/decreased without modifying any other solute-solute or solute-solvent interaction (Methods section). Infrequent WT-MetaD simulations activating monomer de-stacking from a surface hot spot provided Poisson fitting curves^35^ (Methods section) and the characteristic transition timescale for Step 1 (*t*_1_) as a function of ε (Fig. 7b). We can observe that monomer escape from the hot spots becomes slower as the core-core interaction (ε) is progressively strengthened (escape rate: 1/*t*_1_). Shown in Fig. 7b, the residence time onto the hot spot *t*_1_ increases from ~µs (~10^−6^ s) for the original/reference system (solid red curve: ε) to ~10 µs (~10^−5^ s) for the 1.5ε system. This is the same timescale *t*_1_ obtained for Step 1 in fibre (**2**) (Fig. 6 and Supplementary Fig. 5a). *t*_1_ increases up to ~0.1–1 s when augmenting the core-core interaction to 3ε (red dotted curves). In this case, while core stacking presents defects like in the original fibre, monomer escape from the hot spots becomes even slower than jumping in water of a monomer absorbed on the surface (not stacked to any other core in the fibre). Shown in Fig. 7b (blue), Step 2 (*t*_2_) is constant in all cases as this depends on monomer hydrophobicity rather than on the strength of the core-core interaction in this toy model. On the opposite, decreasing the core-core interaction in the model to 0.75ε increases the monomer escape rate from the hot spots (Fig. 7b). In this case the residence time onto the hot spot decreases to *t*_1_ ~10–100 ns (10^−8^–10^−7^ s). Analogous acceleration effect is also obtained, for example, by eliminating the H-bonding between the BTA monomers in our model – *i.e*., by setting to 0 the partial charges explicitly mimicking the H-bonding between the amide beads in our BTA CG models (Fig. 7b: fibre (**1**)_noHB_ in black).

Long (20 µs) CG-MD simulations demonstrate that the spontaneous monomer diffusion between hot spots is way (~100–1000 fold) faster in 0.75ε and (**1**)_noHB_ systems than in 1.5ε (Fig. 7c; Supplementary Fig. 7: data for (**1**)_noHB_), while the average residence time on the hot spots obtained from these unbiased runs is again found consistent with the *t*_1_ timescales obtained via our WT-MetaD approach. The increased dynamics of (**1**)_noHB_ compared to fibre (**1**) also well correlates with recent experiments, showing that lack of H-bonding between the monomers produces more dynamic fibres in water^30^. These results well demonstrate that weakening/strengthening the interactions between the cores (compared to the other interactions in the system) makes the hot spots and, overall, the surface of these fibres more/less dynamic. Seen in Fig. 7d, the spontaneous monomer ∆*z* shifting along fibre 0.75ε (20 µs of CG-MD) is larger compared to system 1.5ε (Supplementary Fig. 7: same data for (**1**)_noHB_). In the same CG-MD time lapse, the monomers in fibre 3ε, including those in the defects, appear as completely static (no monomer surfing along the fibre). This suggests that, in principle, by changing the monomer structure, and tuning the relative strength of the core-core interaction, it is possible to determine the surface dynamics in the fibres.

This toy model is interesting as it allows exploring extreme cases (even far from the real fibres) precluded to the experiments. The obtained results, while clearly qualitative, provide insight into the role played by key factors, such as, for example, the hydrophobicity or the directional interactions in the monomers, on the dynamics of these supramolecular polymers (see also Discussion section).

## Discussion

Studying the dynamics of supramolecular polymers at submolecular resolution is key for building structure-dynamics relationships useful to understand how to customize the monomers to accelerate/slow down the dynamics of supramolecular polymers.

We used WT-MetaD simulations to characterize the mechanism, pathways and kinetics of monomer exchange in atomistic and coarse-grained models of BTA supramolecular polymers. Starting from AA models of simple BTA fibres in the gas phase and in organic solvent, we obtained a molecular-level insight: monomer exchange proceeds via the creation of discontinuities/defects along the stack, while the free-energies associated to this process depend on the level of order into the stack and monomer solubility in the different environments (Fig. 2).

While in these intrinsically ordered conditions the dynamic creation of defects is key to the exchange process, in water BTA supramolecular polymers intrinsically possess a certain number of discontinuities. These are due to the complex interplay of different factors, such as the length/flexibility of the BTA side chains, the competition between directional and non-directional interactions between the BTAs, and their interaction with the solvent (hydrophobicity). These structural defects work as hot spots for monomer exchange (Fig. 3).

Our WT-MetaD simulations reveal that, in water, monomer exchange from the fibre surface is a stepwise process, where monomers diffuse on the surface moving fast from one hot spot to another (Step 1) until finally jumping in water (Step 2) (Supplementary Movies 1, 2). This clearly highlights the dynamic nature of the surface of these fibres. Conversely, exchanging monomers from the interior of the fibre is a more infrequent/unfavourable event, demonstrating that the presence of structural defects (hot spots) along these supramolecular polymers is key for their dynamics. Generalizing our findings, it seems reasonable to hypothesize that when exchange is observed in such supramolecular structures, this originates from defects (hot spots) that are dynamically formed/healed in the assembly. On the contrary, when no exchange is observed, this could be imputable to a too strong interaction between the monomers (*e.g*., hydrophobic, etc.), or high structural perfection within the assembly (lack of exchange hot spots). This structure-dynamics relationship reminds many other cases where defects control the dynamic properties of materials^44–46^, while structural perfection (e.g., diamond) leads to staticity.

Coherent with the experimental evidence^25^, our kinetic analysis shows that, although the mechanism of exchange in and out these fibres is globally similar, each exchange step is slowed down by ~1–2 orders of magnitude in supramolecular polymer (**2**) compared to (**1**). This is imputable to the increased hydrophobicity of (**2**) monomers (Fig. 6c: free-energy necessary for jumping into water doubled respect to (**1**)) and to the increased stacking order and monomer compression into fibre (**2**)^25, 31^.

While our evidences indicate that making the monomer structure more hydrophobic (*e.g*., (**2**) vs. (**1**)) slows down monomer exchange with water (Step 2), in fibres (**1**) and (**2**) this step still depends on the availability of delocalized monomers absorbed on the surface to exchange with the solvent. The dynamics of the monomers present in the surface defects (hot spots) is thus fundamental both for the dynamics of the fibre surface and, in turn, for the exchange with water. We exploited the versatility of our models in the attempt of learning more about how to control the surface dynamics of these fibres.

We observe that by artificially strengthening/weakening the relative strength of the core-core interaction in our fibre models (ε) it is possible to control the residence time of the monomers on the hot spots. On the basis of the results collected from such a toy model we can formulate the following observations. First, accelerating/decelerating of monomer escape from the surface hot spots reflects into a more or less dynamic fibre surface (Fig. 7). In principle, it is thus possible to customize the monomers to augment/reduce the interaction between monomer cores (compared to all other interactions in the system), controlling *de facto* the internal dynamics of these fibres. This is extremely interesting, as surface dynamics in these supramolecular polymers is a key factor for their ability to reorganize their structure in dynamic way in response to external stimuli (e.g., binding to multivalent targets)^23^. Second, the case where the core-core interaction is increased up to 3ε is particularly interesting. In this case, Step 1 (monomer escape from a surface hot spot) is found even slower than Step 2 (monomer exchange with water), becoming the new rate-limiting step in the exchange process (Fig. 7b). In such conditions, the system needs to wait for an available monomer to exchange with the solution—i.e., a monomer that leaves the hot spot remaining absorbed onto the fibre surface. Thus, in principle, by opportunely tuning the balance between key interactions in the system (Step 1 vs. Step 2: core–core vs. hydrophobic, etc.) it could be also possible to control monomer exchange in and out these fibres.

We come out with a submolecular resolution picture of these dynamic supramolecular polymers. Our results clearly demonstrate the bioinspired character of these fibres, where the external skin/surface is continuously renewed while the internal core appears as more static. Moreover, we obtain a detailed insight into the factors that control the rate of monomer exchange within (internal dynamics) and in an out these fibres. Thanks to the advantages of our approach we can gain a molecular-level comprehension of the dynamic nature of these supramolecular polymers that is fundamental to advance our understanding of how to rationally design bioinspired materials with controllable dynamic properties.

## Methods

### AA and CG Models of BTA Fibres and Classical MD Simulations

The AA models for the BTA supramolecular polymers simulated herein have been taken from our previous works, or parametrized accordingly (see Supplementary Information for extended methods)^25, 30^. For the AA BTA-C_6_, we started from pre-stacked 24-mers that have been preliminarily equilibrated via 200 ns of AA-MD as immerged in explicit C_5_ molecules, as well as in the absence of the solvent (gas phase). For the CG models of water-soluble BTA supramolecular polymers, we used our recently developed CG models for monomers (**1**) and (**2**)^31^. These are based on the MARTINI force field^47^, while they also include an explicit treatment of inter-monomer H-bonding^31^. These transferable CG BTA models are consistent with the AA models^30^ for all key factors controlling these supramolecular polymers (behaviour of the monomers in solution, monomer-monomer interactions, cooperativity of H-bonding and self-assembly, amplification of order during supramolecular polymerization, etc.) and with the experimental trends^31^. We started from 80 initially extended pre-stacked (**1**) and (**2**) monomers replicating along the main fibre axis through periodic boundary conditions, modelling the bulk of infinite fibres^25, 30, 31^. CG-supramolecular polymers (**1**) and (**2**) have been preliminarily equilibrated through 6 µs of CG-MD in explicit water. Longer unbiased CG-MD runs (30 µs) were used for the analyses of fibre dynamics (Figs 4 and 6: *t*_3_ and *t*_4_ and Fig. 5).

To better compare the structure of CG water-soluble fibres (**1**) and (**2**) with the BTA-C_6_ oligomers, soluble in organic solvent, we also created an analogous CG model for this system. The CG model for a BTA-C_6_ fibre was built starting from initially extended CG-fibre (**1**), cutting the PEG terminal units and leaving two of the four hydrophobic CG beads of the alkyl chains (Supplementary Fig. 3). As previously done in the development of the CG models for BTA water-soluble monomers^31^, the ±*q* charges in the CG amide beads of CG BTA-C_6_ have been opportunely adjusted to reproduce the correct monomer-monomer dimerization free-energy (Supplementary Methods). As the organic solvent in this CG model we used octane (C_8_), which is a well validated standard in the MARTINI environment^48^. All AA and CG simulations and analyses conducted in this work used the GROMACS 5.1.2 software^49^ and the PLUMED 2 plugin^50^ (see Supplementary Methods for details). The equilibrated configurations for the AA and CG-fibre models served as the input for the WT-MetaD study of monomer exchange.

### Toy models

Taking our CG model for fibre (**1**) as a reference, we used this as a toy model to understand the effect of tuning determined interactions between the monomers (i.e., core-core interaction against monomer hydrophobicity) in these supramolecular polymers. In our CG-fibre model, this was done by progressively strengthening/weakening the interaction between the BTA cores (Fig. 7) without modifying any other solute-solute or solute-solvent interaction (monomer hydrophobicity is unchanged). To this end, we multiplied per 1.5, 3, or 0.75 the interaction strength (ε) between the CG beads composing the aromatic BTA cores (Fig. 7a: original potential well depth for this interaction in these fibres is ε = 0.627 kcal mol^−1^:^31^ Supplementary Methods). We also simulated a case where monomer-monomer H-bonding was deleted ((**1**)_noHB_: see Fig. 7b and Supplementary Fig. 7). This was done by setting to 0 the ±*q* charges explicitly modelling the amide-amide H-bonding in the CG BTA model. All these fibre toy models have been equilibrated, simulated and analyzed as the other CG-fibres (*vide infra*).

### WT-MetaD Study of monomer exchange mechanism and kinetics

Detail on the collective variables (CVs) and WT-MetaD setup used for studying the mechanism of monomer exchange in BTA-C_6_ 24-mers (AA-WT-MetaD) and CG-fibres (**1**) and (**2**) (CG-WT-MetaD) is provided in the Supplementary Methods. The FESs extracted from these CG-WT-MetaD simulations (Figs. 4 and 6) were compared to those obtained using different methods – *i.e*., standard metadynamics^51^ and the method of histograms on 30 µs of unbiased CG-MD. Shown in Supplementary Figs 4a, b, all these techniques produced nearly identical FES.

Monomer exchange with water is a rare event in the timescales effectively accessible using these CG models. Among the various methods used to study rare events in complex molecular systems^32–36, 38–43^, recently it has been reported that the real (unbiased) dynamics of an event (e.g., drug-protein unbinding^33^, etc.^34^) is related to the transition time associated to events activated by infrequent WT-MetaD simulations (biased dynamics)^32, 35^. This approach is particularly convenient as it allows to directly extract information on the kinetics of the activated transition from the biased WT-MetaD simulations. Adapting this approach to our purpose, we calculated the characteristic timescales for all key monomer exchange steps for fibres (**1**) and (**2**) by running multiple infrequent WT-MetaD simulations where the systems undergo transition from **A** to **B** or from **B** to **C** (see Supplementary Methods for details on the CVs and WT-MetaD setup used in these simulations)^32, 35^. The unbiased transition time (*t*) of each transition can be calculated from each WT-MetaD run as:

1 $$t = {t_{{\rm{WT - MetaD}}}}{\left\langle {{{\rm e}^{\beta \left( {V\left( {s\left( {\bf R} \right),t} \right)} \right)}}} \right\rangle _{{\rm{WT}} - {\rm{MetaD}}}}$$

where *V(s(* **R** *),t)* is the time dependent bias, the exponential (brackets) is averaged over the WT-MetaD run and *β* is kT^−1^. The transition times (*t*) calculated from multiple WT-MetaD runs were used to build the transition probability distributions *P*_*n≥1*_:

2 $${P_{n \ge 1}} = 1 - {{\rm e}^{ - \frac{t}{{\rm{\tau }}}}}$$

where *τ* is the characteristic timescale for the various transitions (Figs 4 and 6: *t*_1_: *τ* for **A–B** transition; *t*_2_ for **B–C**, etc.). The *P*_*n≥1*_ distributions were found fitting well with the typical Poisson distributions expected for rare events (see Fig. 7b and Supplementary Fig. 5a), proving the appropriateness of our setup^35^.

While these transition timescales are obtained from simplified CG models, these preserve qualitative value and can be safely used to compare between them and between fibre (**1**) and fibre (**2**) (see below). The fact that *t*_4_ from unbiased CG-MD is found consistent with *t*_1_ from WT-MetaD, and that the difference in *t*_1_ (or *t*_4_) between fibres (**1**) and (**2**) is identical in all cases (~1 order of magnitude, in line with the experimental evidence^25^) demonstrates the correctness of our WT-MetaD approach. Furthermore, we could obtain reliable time distributions for *t*_1_ (**A-B** transition) also using the AA models of these fibres via AA-WT-MetaD simulations. Also at AA-level, *t*_1_ is found slower by ~1 order of magnitude in fibre (**2**) compared to fibre (**1**) (Supplementary Fig. 5b), proving that our CG models can reliably capture kinetic differences between fibres (**1**) and (**2**).

We also activated via WT-MetaD the exchange of monomers stably incorporated in the interior of the fibres, or of dimers absorbed onto the surface of CG-fibre (**1**). These qualitative tests clearly demonstrate that, while possible, both the exchange of monomers from the ordered domains in the fibres or of groups/aggregates of monomers are extremely unfavourable and infrequent events compared to the exchange of monomers from the fibre surface (vide supra, Supplementary Methods and Supplementary Figs 5a and 6 for details).

### Data availability

The data sets generated and analyzed during the current study are available from the corresponding author on reasonable request.

## Electronic supplementary material

Supplementary Information

Supplementary Movie 1

Supplementary Movie 2

Peer Review
