# Supplementary material for: Into the Dynamics of a Supramolecular Polymer at Submolecular Resolution
Source: Nat Commun. 2017 Jul 27;8:147. doi: 10.1038/s41467-017-00189-0 (PMC5529520; doi:10.1038/s41467-017-00189-0)
Supplement: Supplementary file 1 — Supplementary Information [file 41467_2017_189_MOESM1_ESM.pdf]

File Name: Supplementary Information
Description: Supplementary Figures, Supplementary Tables, Supplementary Methods and Supplementary References

File Name: Supplementary Movie 1
Description: Mechanism of monomer exchange in a water-soluble BTA supramolecular polymer – Lateral chains of BTA fibre 1 are represented as transparent, BTA cores in green and the activated BTA core is represented as a red dot (water not shown for clarity).

File Name: Supplementary Movie 2
Description: Monomer surfing on the surface of a supramolecular fibre – Monomers in Fibre 1 are colored based on their position along the main fibre axis (z-displacement: blue-white-red), the surfing monomer is identified in green (terminal PEG units and water not shown for clarity).

File Name: Peer Review File
Description:

Supplementary Methods

Creation of the AA and CG models, classical MD simulations and analysis of the BTA fibres

The AA models for water-soluble BTA monomers (**1**) and (**2**) (**Figure 1a**) have been reported in our previous works.[1-2] The AA models for BTA-C_6 monomers were built and parametrized accordingly[2] based on the general AMBER force field (GAFF) (*gaff.dat*).[3] We created initially extended AA BTA-C_6 24-mers, where the monomer cores were pre-stacked at intercore distance of 3.4 Å.[1-2] The 24-mers were then inserted in a simulation box filled with explicit C_5 solvents molecules, as well as in the absence of solvent (gas phase).

All MD simulations in this work were conducted with the GROMACS 5.1.2 software.[4] The AA BTA-C_6 24-mers were preliminarily minimized and heated to reach the temperature of 300 K (27 °C). Then, all AA systems underwent 200 ns of classical MD simulations in NPT conditions, using a timestep of 2 fs and a 10 Å cutoff. During this time, all BTA-C_6 24-mers successfully reached the equilibrium in the AA-MD regime. All equilibration AA-MD runs were conducted at 27 °C using the v-rescale[5] thermostat (coupling constant of 2.0 ps). Isotropic pressure scaling kept the pressure in the system at 1 atm with coupling constant of 2 ps. We used the particle mesh Ewald (PME)[6] approach to treat long-range electrostatics and the LINCS algorithm to constrain all bonds involving hydrogens.[7] The equilibrated configurations obtained for the 24-mers in the different environments served as the input for the WT-MetaD study of monomer exchange (see below).

The AA models for water-soluble BTA supramolecular polymers (**1**) and (**2**) resulted too complex to study monomer exchange in accurate way. Recently, we developed CG models for water-soluble BTA monomers (**1**) and (**2**) (**Figure 1a**) based on the MARTINI force field,[8] which also include the explicit treatment of inter-monomer H-bonding via rigid rotating dipoles introduced into the CG beads for the BTA amide groups.[9] These CG models demonstrated optimal consistency with the experimental trends, and with the AA models of the same BTAs,[2] correctly capturing all key factors controlling these supramolecular polymers – *i.e.*, behavior of the monomers in solution, monomer-monomer interactions, cooperativity of H-bonding and self-assembly, and amplification of order into the growing fibres during supramolecular polymerization.[9]

The CG models for water-soluble BTA fibres (**1**) and (**2**) simulated in this work were obtained starting from 80 initially extended (**1**) and (**2**) monomers perfectly stacked and inserted into a periodic simulation box filled of standard W MARTINI water beads.[9] The simulation box was built grazing the terminal BTA cores along the main fibre axis, effectively modeling via periodic boundary conditions the bulk of infinite BTA fibres surrounded by water. The CG models for fibres (**1**) and (**2**) have been equilibrated via classical CG-MD simulations in NPT conditions (constant N: number of particles, P: pressure and T: temperature during the run) for 6 μs using a timestep of 20 fs. All equilibration CG-MD runs were conducted at 27 °C using the v-rescale[5] thermostat with coupling constant of 2.0 ps. Consistent with the directional nature of these infinite CG fibre models, semi-isotropic pressure scaling was used to keep the pressure in the system at 1 atm (coupling constant of 8 ps).[9] A straight cutoff (1.1 nm) and potential modifiers combined with the Verlet neighbor list scheme[10] were used in all CG simulations. Equilibrated fibres (**1**) and (**2**) served as the input for the WT-MetaD study of monomer exchange and the analysis of the fibre dynamics (see below).

Order/disorder analyses to identify the hot spots along the fibres were conducted on the equilibrated phase CG-MD trajectories using the GROMACS utilities *gmx_sasa* (for SASA), *gmx_energy* (for ΔE) and the PLUMED 2 plugin (coordination number: coordination between the cores of the monomers).[11] In **Figures 3f,6a** the latter was put in relation with the SASA of each monomer expressed in terms of percentage variation from the average (ΔSASA(%)). This allows comparing between different fibres (different size monomers) and to unambiguously identify the hot spots in the fibres (monomers with coordination ≤1 and SASA larger than the average: colored monomers in **Figures 3f,6a**). The AA model for fibre (**1**) shows analogous discontinuities/defects along the fibre that are spontaneously formed even after a few hundreds of nanoseconds of AA-MD simulation (**Supplementary Figure 2**).[1,2] Considering that such AA-MD runs start from a perfectly extended configuration for the BTA fibre and the limited sampling that can be achieved during these simulations (compared to CG simulations), this demonstrates that the formation of these defects (hot spots) is intrinsically favored in these BTA stacks in water (and not somehow due to the CG models).

To better compare the structure of CG water-soluble fibres (**1**) and (**2**) with BTA-C_6 ones, soluble in organic solvent, we also created an analogous CG model for this system. The CG model for BTA-C_6 monomers was built cutting the PEG terminal units and two of the four hydrophobic CG beads of the alkyl groups from the side chains of CG BTA monomer (**1**) (see **Supplementary Figure 3a**). As the organic solvent in this CG model we used octane (C_8), which is a well-validated standard in the MARTINI environment. According to the same protocol previously adopted for the development of the CG models of BTA water-soluble monomers,[9] the value of the $\pm q$ explicit charges in the CG amide beads of CG BTA-C_6 monomers has been opportunely adjusted (to $\pm q = \pm 0.8\ e$) to reproduce the correct dimerization free-energy for the same monomers in the same conditions at AA level ($\Delta G = -10.7 \pm 1$ kcal mol^{-1}, calculated via metadynamics simulations). An initial configuration for the BTA-C_6 fibre in organic solvent was obtained from that of CG fibre (**1**) composed of 80 initially extended monomers replicating along z-direction through periodic boundary conditions. The side chains of fibre (**1**) were cut and the simulation box was filled of C_8 solvent molecules (details for all simulated systems are reported in **Supplementary Table 1**). BTA-C_6 fibre in C_8 has been simulated for 10 μs of CG-MD using the same simulation protocol adopted for all other CG fibres described above. This simulation time was sufficient for full equilibration, as this fibre is more static than water-soluble ones. The same analyses conducted for water-soluble fibres reveal no presence of hot spots in this ordered stack (see **Figure 3f**: purple), where all monomers lie around the average SASA and ΔE values as it is expected for a nearly perfect stacking (coordination ~2, see **Supplementary Figure 3**).

Analyses of the minimum distance and coordination between the initially dissolved monomer and the cores in the equilibrated fibres (**Figure 5a,b**) were conducted with the PLUMED 2 plugin[11] on 30 μs NPT CG-MD simulations of fibres (**1**) and (**2**) under the same conditions described above. In this case the coordination threshold was set to a value equaling the half of the average distance between the hot spots on the fibres surface (~2 nm from each other). In this way, every time that the coordination drops from 1 to 0 (**Figure 5a,b**: red or blue) the monomer leaves the hot spot and moves to a new one. Analysis of the Δz shifts and average monomer drift velocity ($\Delta \bar{v}_z$) of the individual monomers along the fibres (**Figures 5d-f**) were conducted on the last 20 μs of CG-MD simulations using the *gmx_distance* tool of GROMACS. The $\Delta \bar{v}_z$ data

(**Figure 5d**) were obtained summing the Δz (absolute values) for the individual monomers calculated every 300 ns, and grouping the monomers based on their distance from the fibre centre.

Toy models

Taking CG fibre (**1**) as a reference, we used the latter as a toy model where we progressively strengthened/weakened the interaction between the BTA cores (**Figure 7**) without modifying any other solute-solute or solute-solvent interaction (monomer hydrophobicity is preserved). To this end, we multiplied per 1.5, 3 or 0.75 the interaction strength (ε) between the CG beads (SC5) composing the aromatic BTA cores (**Figure 7a**: original potential well depth for SC5-SC5 interaction in these CG fibres: ε = 0.627 kcal mol^{-1}).[9] An additional system was modeled where the H-bonding between the monomers was deleted while keeping the original core-core interaction (ε) constant (system $(\mathbf{1})_{noHB}$). This was done by simply setting to 0 the $\pm q$ charges explicitly modeling the amide-amide H-bonding in our CG BTA models.[9] All these fibre toy models were equilibrated, simulated and analysed as the other CG fibres (*vide supra*).

WT-MetaD simulations – monomer exchange in BTA stacks in gas phase and organic solvents

All WT-MetaD[12] simulations have been conducted using the GROMACS 5.1.2 software[4] and the PLUMED 2 plugin.[11] We found that the minimum distance (CV1) and the coordination between the core of the activated monomer (**Figure 2a**: red) and the closest neighbors in the stack (CV2) work well as the collective variables (CVs) describing/biasing monomer exchange from the centre of the BTA-C_6 24-mers. The intrinsically ordered nature of these BTA stacks in the gas phase and in organic solvent facilitates these *in silico* experiments, as choosing a different monomer from the fibre center has negligible effect on the outcomes. In both environments, the activated monomers were seen to leave the oligomers and exchange during the WT-MetaD runs (snapshots in **Figures 2a**), being subsequently reincorporated into the stack (recrossing). For the AA-WT-MetaD runs, we used a HILLS height to 0.6 kcal mol^{-1}, and a Gaussian SIGMA of 0.05 nm (gas phase) or 0.01 nm (C_5 and C_9 solvents) for CV1 and 0.1 for CV2. From the AA-WT-MetaD simulations (1.5 μs) we obtained the free-energy surfaces (FESs) for monomer exchange in the different environments reported in **Figures 2b,c**.

WT-MetaD simulations – mechanism of monomer exchange in BTA water-soluble fibres

Starting from equilibrated configurations for CG-fibres (**1**) and (**2**) obtained from the CG-MD (*vide supra*), we focused on the study of monomer exchange from the surface hot spots. The minimum distance (CV1) between the core of the activated monomer (**Figure 4d**: green) and the closest neighbor in the hot spot (**Figure 4d**: red), and the distance from the fibre surface calculated on the plane perpendicular to the main axis of the fibre (CV2) demonstrated to be well suited CVs to describe/activate the exchange with water.

The event of monomer exchange with water was found too complex for reaching satisfactory convergence during a single CG-WT-MetaD run. Thus, we first conducted multiple explorative CG-WT-MetaD simulations biasing the extraction of the activated monomer from the fibre to understand the general mechanism. Averaging ten extraction CG-WT-MetaD runs provided a reliable qualitative free-energy profile for the monomer exchange process (**Figure 4a**: represented as a function of CV1). This shows two distinct main steps in monomer exchange with water (**Figure 4a**: Step 1: **A-B** transition and Step 2: **B-C** transition), while this observation did not change activating the exchange of another monomer from a different hot spot. Building on this clear evidence, we then focused on the individual **A-B** and **B-C** transitions, for which we could obtain quantitative FESs. Good convergence FESs for individual Step 1 and Step 2 (**Figure 4b,c**) were obtained using respectively only CV1 (HILLS height of 0.024 kcal mol^{-1}, Gaussian SIGMA of 0.01 nm, deposition time of 20 ps and bias factor of 8) and CV2 (HILLS height of 0.12 kcal mol^{-1}, Gaussian SIGMA of 0.04 nm, deposition time of 10 ps and bias factor equal to 25) in the WT-MetaD runs, and recrossing multiple times between the involved states (see **Supplementary Figure 4**).

We challenged these WT-MetaD results by using different methods. Standard metadynamics[13-14] using the same CVs of WT-MetaD and recrossing between **A-B** and **B-C** states produced analogous FESs. The **A-B** transition (Step 1) is relatively fast in CG-fibre (**1**). Thus, we could obtain an analogous FES also from 30 μs of unbiased CG-MD with the method of histograms. Shown in **Supplementary Figures 4a,b**, all these techniques produced nearly identical FES for Steps 1 and 2.

WT-MetaD simulations – kinetics of monomer exchange in BTA water-soluble fibres

Within the timescales that can be effectively explored using these CG models, the exchange of a monomer with water is a rare event. Various computational approaches have been used to study rare events in complex molecular systems (*e.g.*, Monte-Carlo to study nucleation,[15-16] replica MD and Markov state models to study protein conformational plasticity and drug binding kinetics,[17] to name a few). Recently, it has been reported that the mechanism and kinetics of drug unbinding from a protein binding pocket,[18] and other rare events,[19-20] can be efficiently modeled by using infrequent WT-MetaD simulations. Inspired by hyperdynamics[21] and conformational flooding,[22] this approach is based on the concept that the real (unbiased) dynamics of an event is related to the transition time associated to activated events (WT-MetaD biased dynamics).[23-24] We used this concept to calculate the relative rates (timescales) for the monomer exchange steps identified in our WT-MetaD simulations.

The characteristic timescales for the individual key steps in monomer exchange (Steps 1 and 2) for fibres (**1**) and (**2**) were calculated from the fit of the unbiased transition times distributions constructed from multiple infrequent WT-MetaD runs where the systems were biased to undergo transition from **A** to **B** or from **B** to **C**.[20-21] The unbiased transition time (t) can be calculated from each individual WT-MetaD run as:

$$t = t_{WT-MetaD} \langle e^{\beta(V(s(\mathbf{R}),t))} \rangle_{WT-MetaD} \qquad (1)$$

where $V(s(\mathbf{R}),t)$ is the time dependent bias, the exponential (brackets) is averaged over the WT-MetaD run and β is kT^{-1}. The transition times (t) calculated from multiple WT-MetaD runs for the **A**-**B** and **B**-**C** transitions were used to build the transition probability distribution $P_{n\geq 1}$ (probability to observe at least one transition by time t). Shown in **Supplementary Figure 5** and **Figure 7** (for the toy models), the collected time distributions were found to fit well with the typical Poisson distributions expected for rare events:[24]

$$P_{n\geq 1} = 1 - e^{-\frac{t}{\tau}} \qquad (2)$$

where τ is the characteristic time for the various transitions (**Figures 4d,6d**: t_1: τ for the **A-B** transition; t_2 for **B-C**, etc.). This proves the appropriateness of the WT-MetaD setup used to calculate the relative kinetics of the observed transitions.[24] While these transition timescales (**Figures 4,6**; **Supplementary Figure 5a**) are

collected from simplified CG models, these still maintain qualitative value and can be used to safely compare fibre (**1**) to fibre (**2**) (see also below: comparison to AA, **Supplementary Figure 5b**).

For the calculation of t_1 we used the same setup (CV1) used for the FES calculation (*vide supra*), decreasing the HILLS deposition time to 40 ps. Conversely, for the characteristic timescale (t_2: **B**-**C** transition), we used as CV2 the number of contacts between the hydrophobic CG beads of the activated monomer and those of the other monomers in the fibres, using a cutoff for contacts search of 1 nm (HILLS height of 0.12 kcal mol^{-1}, Gaussian SIGMA of 0.015, deposition time of 100 ps and bias factor of 25). This CV2 provided better Poisson distributions for the timescales of monomer exchange with water, and facile interpretation of the transition (contacts drop rapidly to 0 while jumping into water).

We also calculated the characteristic transition timescale (t_0) for dragging a monomer stably incorporated in the fibre interior to the surface (Step 0: using CV1 in the WT-MetaD runs). Considering the statistic/dynamic, non-perfect nature of these fibres, t_0 constitutes a qualitative upper limit for monomer exchange, while the t_0 value may vary depending on the monomer and, particularly, on the mechanical deformations of the fibres during the simulations that can facilitate the dynamic creation of local defects along the stack. Nonetheless, the striking difference seen between the characteristic timescales from WT-MetaD (**Supplementary Figure 5**) indicate that exchanging monomers with water from the fibre interior (Steps 0-2) is way slower than exchanging monomers already present on the fibre surface (Step 2 only: exchange of monomers absorbed on the surface; Steps 1-2: exchange of monomers from a surface hot spot).

The fact that t_4 from CG-MD is found consistent with t_1 from WT-MetaD, and that the difference in t_1 (or t_4) between fibres (**1**) and (**2**) is identical in all cases (~1 order of magnitude) demonstrates the reliability of our WT-MetaD approach to correctly capture kinetic differences between the two fibres. To further prove that our CG models did not exaggerate or underestimate kinetic differences between the fibres, we could obtain reliable time distributions for t_1 (**A-B** transition) also from AA models of fibres (**1**) and (**2**) via AA-WT-MetaD simulations. The collected time distributions fit well with Poisson ones. The ratio between t_1 in AA-fibres (**1**) and (**2**) was found identical to that obtained using our CG models (**Supplementary Figure 5b**: t_1

slower by ~1 order of magnitude in (**2**) compared to (**1**)), proving that our CG models can reliably capture kinetic differences between fibres (**1**) and (**2**).

Preliminary CG-WT-MetaD tests showed that exchanging groups/aggregates of monomers from the same hot spot is an unlikely event, as the stacked aggregate, deeply incorporated into the fibre, requires fibre breakage to exchange. However, monomer aggregates could in principle also form occasionally on the surface of the fibre via collision between diffusing monomers. Thus, we activated the exchange of an absorbed BTA dimer from the surface of fibre (**1**) via infrequent WT-MetaD, using CV2 (hydrophobic contacts between the dimer and the fibre) as the collective variable and the same setup used to study monomer jumping in water. While pertaining to an extremely complex case, these qualitative tests demonstrate that, although possible, this event is extremely unfavorable and infrequent compared to the exchange of monomers from the fibre surface (see **Supplementary Figure 6**).

Supplementary Table 1: Details of the molecular systems built and simulated in this work

System	# of BTA	Model	Solvent	# of solvent molecules	# of atoms (CG beads)	Total simulation time[a]	# of WT-MetaD runs (kinetics)[b]
BTA-C_6	24	AA	-	-	1872	1.7 μs	-
BTA-C_6	24	AA	C_5	1253	23173	1.7 μs	-
BTA-C_6	80	CG	C_8[c]	9136	20000	10 μs	-
BTA (**1**)	80	CG	Water[d]	20917[d]	24037	125 μs	63
BTA (**2**)	80	CG	Water[d]	20917[d]	24037	120 μs	51
0.75ε[e]	80	CG	Water[d]	20917[d]	24037	20.2 μs	20
1.5ε[e]	80	CG	Water[d]	20917[d]	24037	21 μs	20
3ε[e]	80	CG	Water[d]	20917[d]	24037	28 μs	20
$(\mathbf{1})_{noHB}$[f]	80	CG	Water[d]	20917[d]	24037	20.2 μs	20

[a]Total simulation times including all biased and unbiased simulations conducted for all AA and CG simulated systems. [b]Number of infrequent CG-WT-MetaD runs used to build the Poisson time distributions for Steps 0, 1 and 2 of **Supplementary Figure 5** and **Figure 7b**. [c]As the organic solvent to simulate CG BTA-C_6 we used octane (C_8).[25] [d]All CG models for water-soluble fibres used standard MARTINI W water CG beads.[9] [e]Toy models where the core-core interaction strength was systematically increased/decreased (n*ε) respect to the original (CG fibres (**1**) and (**2**): ε = 0.627 kcal mol^{-1}).[9] [f]Toy model where H-bonding between the BTAs[9] was deleted ($\pm q$=0 in the CG amide beads).

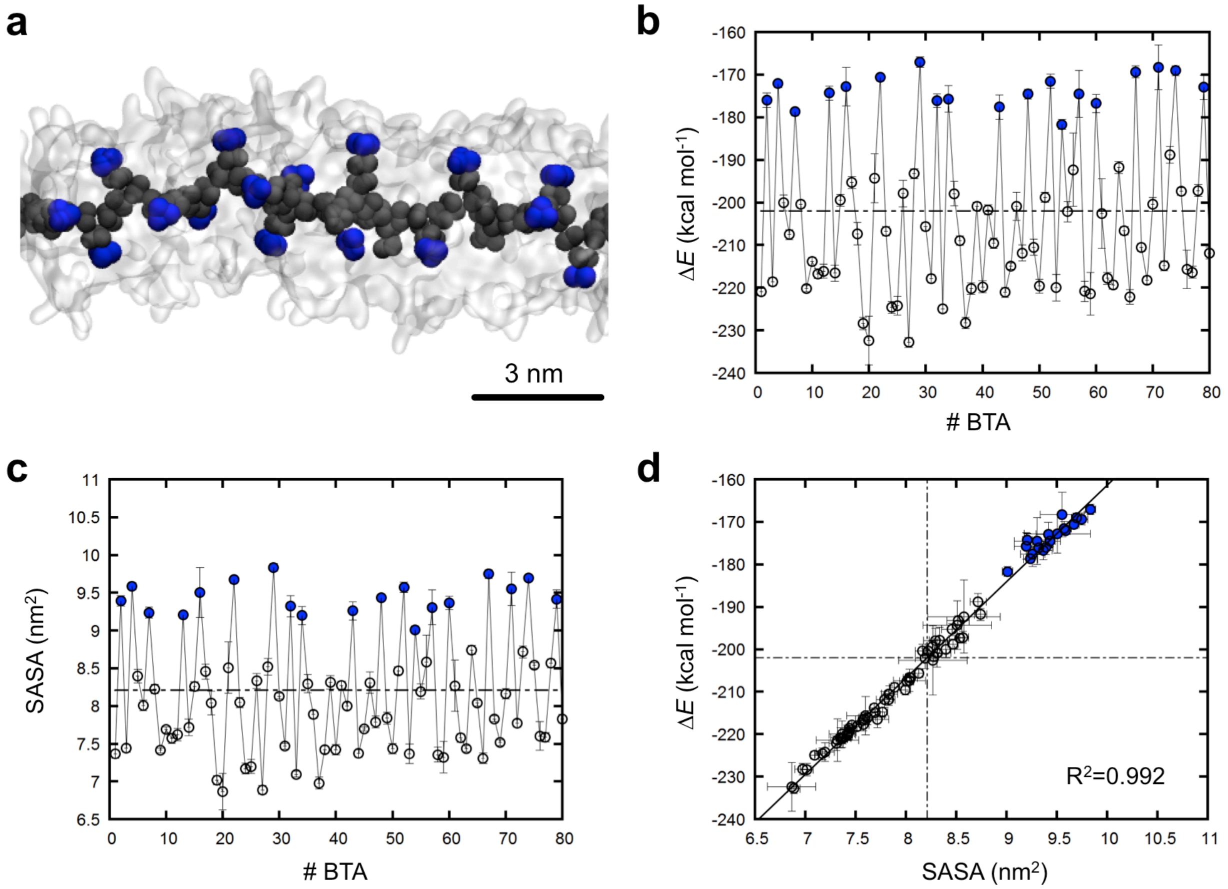

Supplementary Figure 1: Analysis of order/disorder reveals exchange hot spots in an equilibrated CG model of water-soluble BTA supramolecular polymer (2).[9] (a) Equilibrated configuration of CG-fibre (2) in water obtained via CG-MD simulation – BTA cores are shown in dark grey (and blue), the amphiphilic branches of water-soluble monomers (2) are shown in transparent grey (water beads not shown for clarity). (b) Interaction energy (ΔE) of each individual monomer with the rest of CG-fibre (2). (c) Solvent accessible surface area (SASA) for each monomer in equilibrated CG-fibre (2). (d) Inverse linear relationship between the strength of incorporation (ΔE) and monomer exposure to the solvent (SASA). Hot spot monomers are colored in blue. Error bars in the plots represent s.e.m.

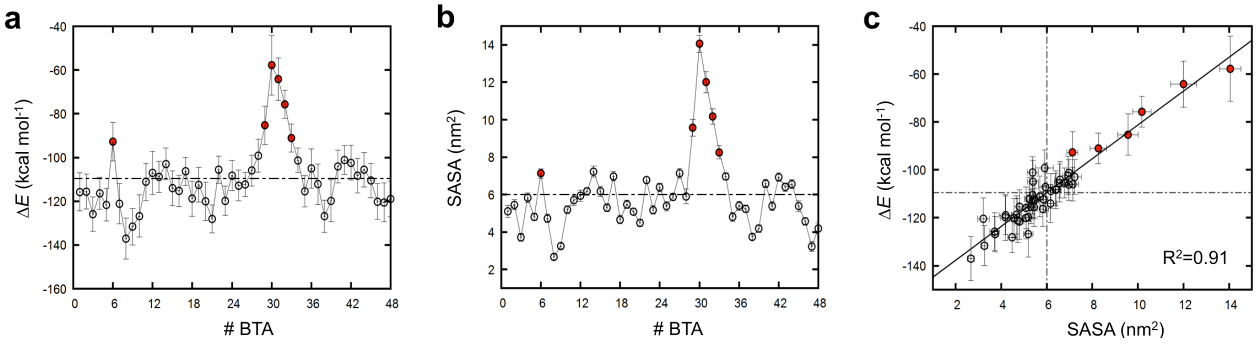

Supplementary Figure 2: Analysis of order/disorder reveals defects (hot spots) in AA-fibre (1).[1-2] (a) Interaction energy (ΔE) of each monomer with AA-fibre (1). (b) Solvent accessible surface area (SASA) for AA monomers. (c) Inverse linear relationship between ΔE and SASA. Hot spot monomers (less favorable ΔE and larger SASA than the average) are in red. While AA models are limited compared to CG ones in the sampling and timescales that can be simulated, these AA results prove that defects (hot spots) are spontaneously formed along these fibres at all levels in water. Error bars in the plots represent s.e.m.

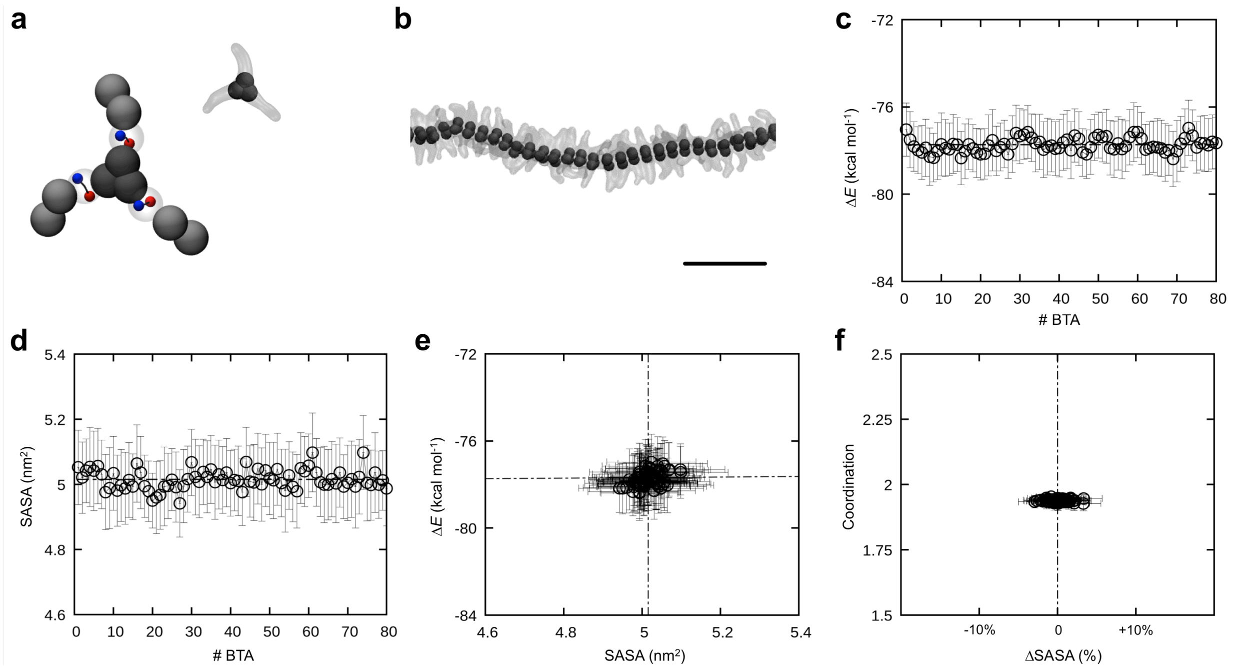

Supplementary Figure 3: Analysis of order in a CG BTA-C$_6$ fibre in organic solvent (C$_8$). (a) CG BTA-C$_6$ monomer model. (b) Equilibrated configuration (10μs of CG-MD) of a CG BTA-C$_6$ fibre (scale bar: 3 nm). (c) Interaction energy (ΔE) of each BTA-C$_6$ monomer with the rest of the CG fibre. (d) Solvent accessible surface area (SASA) for each monomer in CG BTA-C$_6$ fibre. (e) ΔE *vs.* SASA for the monomers in the CG BTA-C$_6$ fibre. Hot spot monomers are colored in blue. (f) Coordination number *vs.* SASA (% deviation from the average) for the individual monomers in CG BTA-C$_6$ fibre in C$_8$. All monomers have coordination ~2, which demonstrates the nearly perfect nature of this fibre in organic solvent (see also Figure 3f). Error bars in the plots represent s.e.m.

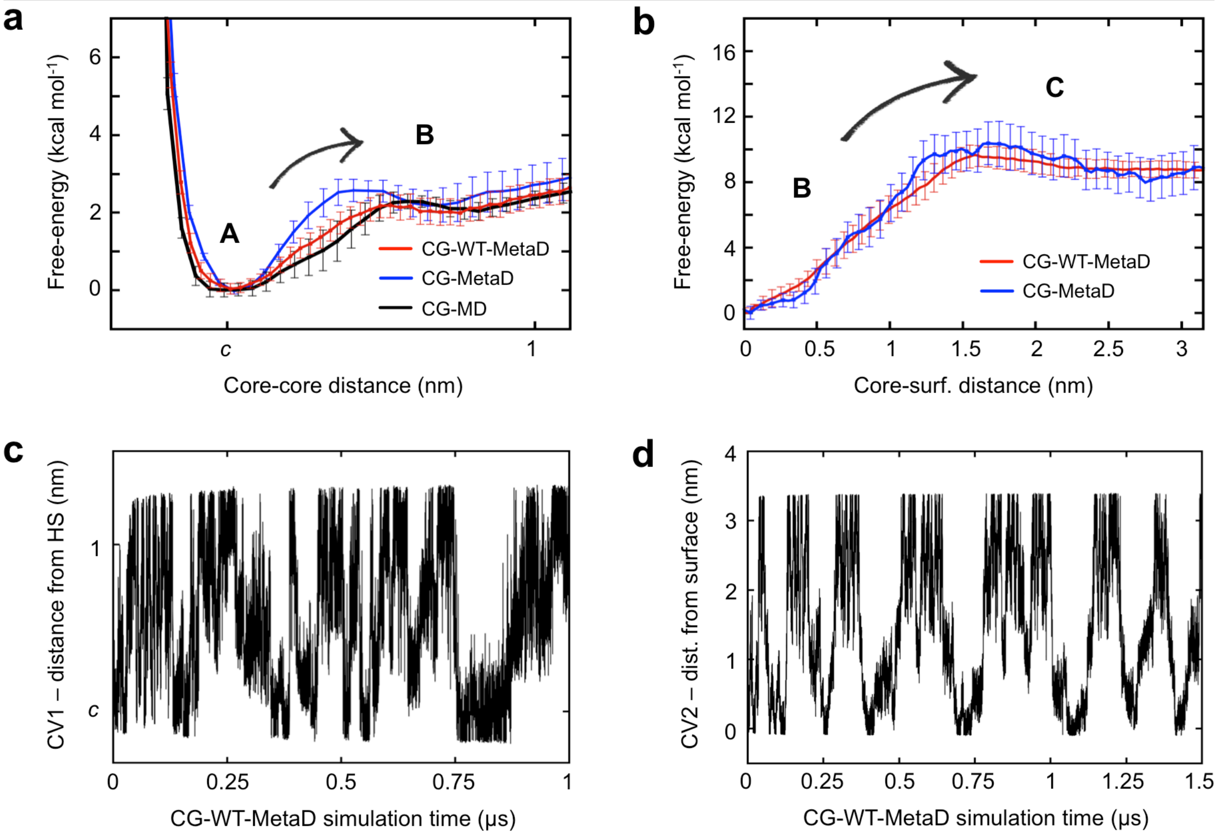

Supplementary Figure 4: FESs for monomer exchange Step 1 and Step 2 in CG-fibre (1). (a) Step 1 FES (A-B transition) as a function of the distance between the activated core and the closest neighbor in the hot spot (CV1). The FES has been calculated via WT-MetaD (red), standard metadynamics (MetaD: blue) and long (30 μs) unbiased CG-MD (black: method of histograms). (b) FES for Step 2 (B-C transition) as a function of the distance between the activated core and the surface of the fibre (CV2). Error bars represent s.e.m. (c,d) Each FES has been calculated by recrossing multiple times between the different states. CV1 (c) and CV2 (d) in time.

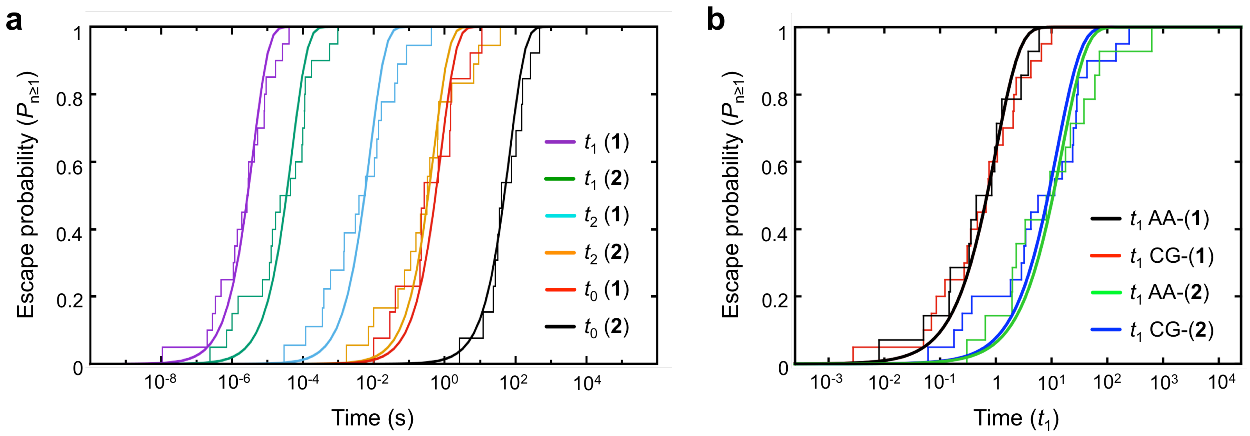

Supplementary Figure 5: Transition time distributions for the monomer exchange steps in CG-fibres (1) and (2) obtained through multiple infrequent WT-MetaD simulations (each segment in the distributions identifies one WT-MetaD run). (a) Extracted transition times fitted to Poisson distributions, obtaining characteristic timescales t_1, t_2 and t_0 for the different steps in monomer exchange for CG-fibres (1) and (2). While the timescales are extracted from CG models and have qualitative value, these are useful to compare between them and between fibres (1) and (2). (b) Transition times t_1 in fibre (2) and fibre (1) obtained from both AA and CG models for these fibres. Both AA and CG models show that t_1 is ~1 order of magnitude slower in fibre (2) than in fibre (1), demonstrating that our CG models can accurately compare between fibres (1) and (2) kinetics.

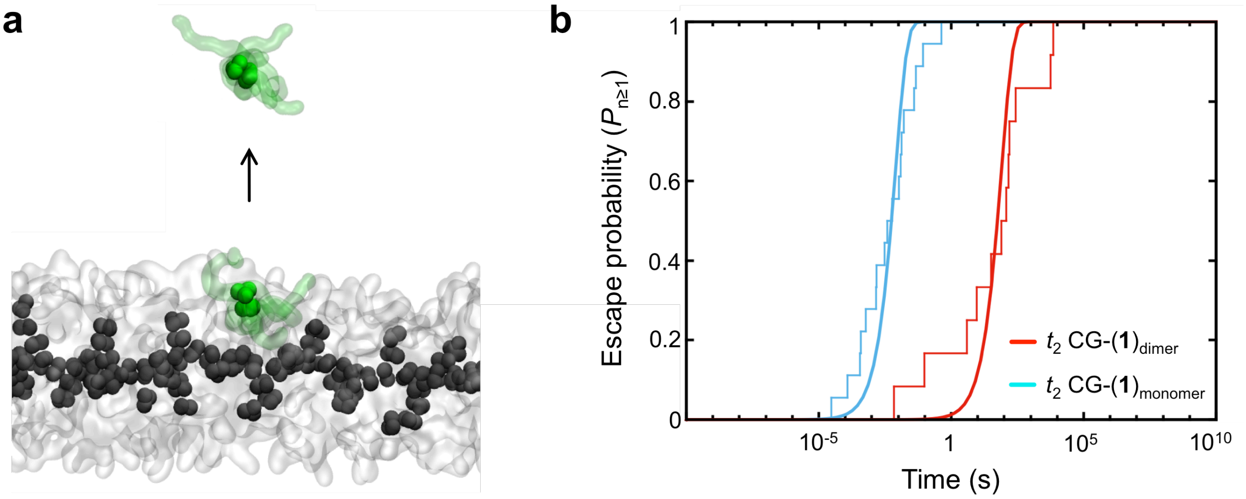

Supplementary Figure 6: Qualitative comparison between the exchange of dimers *vs.* monomers absorbed on the surface of fibre (1). (a) Snapshots from the CG-WT-MetaD simulations biasing/activating the exchange of an absorbed dimer (green) (water beads not shown for clarity). (b) Despite the complexity of this case, we could build a qualitative transition time distribution for dimer exchange (red). The transition time distribution for monomer exchange from the surface of fibre (1) is also reported for comparison (cyan).

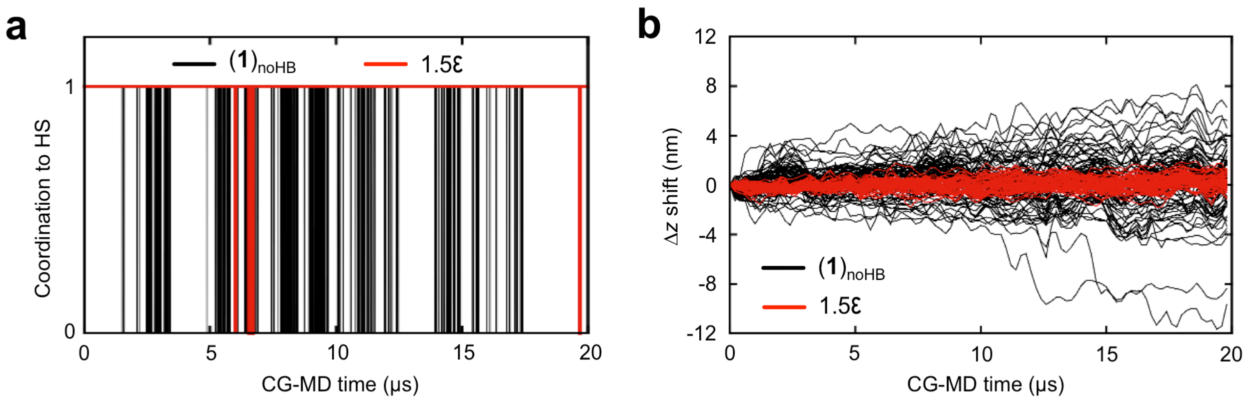

Supplementary Figure 7: Accelerated surface dynamics in toy model $(1)_{noHB}$. (a) Spontaneous monomer diffusion (CG-MD) between the surface hot spots: monomer coordination in $(1)_{noHB}$ system (black). (b) Monomer shifting (Δz) along the main fibre axis in $(1)_{noHB}$ system (black) . Data for system 1.5ε are reported (in red) for comparison.

Supplementary References

1. Baker, M. B. *et al.* Consequences of chirality on the dynamics of a water-soluble supramolecular polymer. *Nat. Commun.* **6**, 6234 (2015).

2. Garzoni, M. *et al.* Effect of H-bonding on order amplification in the growth of a supramolecular polymer in water. *J. Am. Chem. Soc.* **138**, 13985–13995 (2016).

3. Wang, J., Wolf, R. M., Caldwell, J. W., Kollman, P. A. & Case, D. A. Development and testing of a general amber force field. *J. Comput. Chem.* **25**, 1157–1174 (2004).

4. Abraham, M. J. *et al.* Gromacs: High performance molecular simulations through multi-level parallelism from laptops to supercomputers. *SoftwareX* ***1–2***, 19–25 (2015).

5. Bussi, G., Donadio, D. & Parrinello, M. Canonical sampling through velocity rescaling. *J. Chem. Phys.* **126**, 14101 (2007).

6. Darden, T., York, D., Pedersen, L. Particle mesh Ewald: An *N*·log(*N*) method for ewald sums in large systems. *J. Chem. Phys.* **98**, 10089-10092 (1993).

7. Hess, B., Bekker, H., Berendsen, H. J. C. & Fraaije J. G. E. M. LINCS: A linear constraint solver for molecular simulations. *J Comput Chem.* **18**, 1463–1472 (1997).

8. Marrink, S. J., Risselada, H. J., Yefimov, S., Tieleman, D. P. & De Vries, A. H. The MARTINI force field: Coarse grained model for biomolecular simulations. *J. Phys. Chem. B* **111**, 7812–7824 (2007).

9. Bochicchio, D. & Pavan, G. M. From cooperative self-assembly to water-soluble supramolecular polymers using coarse-grained simulations. *ACS Nano.* **11**, 1000–1011 (2017).

10. De Jong, D. H., Baoukina, S., Ingólfsson, H. I. & Marrink, S. J. Martini straight: Boosting performance using a shorter cutoff and GPUs. *Comput. Phys. Commun.* **199**, 1–7 (2016).

11. Tribello, G. A., Bonomi, M., Branduardi, D., Camilloni, C. & Bussi, G. PLUMED 2: New feathers for an old bird. *Comput. Phys. Commun.* **185**, 604–613 (2014).

12. Barducci, A., Bussi, G. & Parrinello, M. Well-tempered metadynamics: A smoothly converging and tunable free-energy method. *Phys. Rev. Lett.* **100**, 020603 (2008).

13. Laio, A. & Parrinello, M. Escaping free-energy minima. *Proc. Natl. Acad. Sci. U. S. A.* **99**, 12562–12566 (2002).

14. Barducci, A., Bonomi, M. & Parrinello, M. Metadynamics. *Wiley Interdiscip. Rev. Comput. Mol. Sci.* **1**, 826–843 (2011).

15. Auer, S. & Frenkel, D. Prediction of absolute crystal-nucleation rate in hard-sphere colloids. *Nature* **409**, 1020-1023 (2001).

16. Valeriani, C., Sanz, E. & Frenkel, D. Rate of homogeneous crystal nucleation in molten NaCl. *J. Chem. Phys.* **122**, 194501 (2005).

17. Plattner, N. & Noé, F. Protein conformational plasticity and complex ligand-binding kinetics explored by atomistic simulations and Markov models. *Nat. Commun.* **6**, 6753 (2015).

18. Tiwary, P., Limongelli, V., Salvalaglio, M. & Parrinello, M. Kinetics of protein-ligand unbinding: Predicting pathways, rates, and rate-limiting steps. *Proc. Natl Acad. Sci. USA* **112**, E386 (2015).

19. Salvalaglio, M., Tiwary, P., Maggioni, G. M., Mazzotti, M., Parrinello, M. Overcoming time scale and finite size limitations to compute nucleation rates from small scale well tempered metadynamics simulations. *J. Chem. Phys.* **145**, 211925, (2016).

20. Lelimousin, M., Limongelli, V. & Sansom, M. S. P. Conformational changes in the epidermal growth factor receptor: Role of the transmembrane domain investigated by coarse-grained metadynamics free energy calculations. *J. Am. Chem. Soc.* **138**, 10611-10622 (2016).

21. Voter, A. F. Hyperdynamics: Accelerated molecular dynamics of infrequent events. *Phys. Rev. Lett.* **78**, 3908 (1997).

22. Grubmüller, H. Predicting slow structural transitions in macromolecular systems: Conformational flooding. *Phys. Rev. E* **52**, 2893 (1995).

23. Tiwary, P. & Parrinello, M. From metadynamics to dynamics. *Phys. Rev. Lett.* **111**, 230602 (2013).

24. Salvalaglio, M., Tiwary, P. & Parrinello, M. Assessing the reliability of the dynamics reconstructed from metadynamics. *J. Chem. Theory Comput.* **10**, 1420-1425 (2014).

25. Marrink, S. J., De Vries, A. H. & Mark, A. E. Coarse Grained model for semiquantitative lipid simulations. *J. Phys. Chem. B* **108**, 750-760 (2004).
