# Supplementary material for: Into the Dynamics of a Supramolecular Polymer at Submolecular Resolution
Source: Nat Commun. 2017 Jul 27;8:147. doi: 10.1038/s41467-017-00189-0 (PMC5529520; doi:10.1038/s41467-017-00189-0)
Supplement: Supplementary file 4 — Peer Review [file 41467_2017_189_MOESM4_ESM.pdf]

Reviewers' comments:

Reviewer #1 (Remarks to the Author):

This is an interesting manuscript which reports mechanistic, thermodynamic and dynamical details of monomer binding/unbinding transitions to a supramolecular oligomer. The work is carried out using MD simulations, and follows a previously published experimental work using the STORM technique. The simulations are carried out competently and the analyses are exquisitely done. Detailed insights are provided. The result shown in Figure 2 in particular is quite good & illuminating. In its current form, the manuscript is not recommended for publication. However, in order to proceed further on a recommendation, some vital concerns need to be addressed. These are:

1) What is the purpose of reporting simulations of the BTA-C6 in C5? (Figure 2). How does it fit into the rest of the paper? This is unclear in the current MS.

2) Figure 2 are results from AA simulations in an organic solvent of a supramolecule which is largely hydrophobic, while the rest of the MS discusses hydrophilic polymers in water, modelled using coarse grained force field. On page 5 (one sentence prior to the section on "Intrinsic dynamics ..."), the authors state that water soluble fibers possess hot spots (there appears to be an allusion that the water soluble fibers have more hot spots than the C5 soluble ones, but the sentence is quite unclear). What is the evidence for this allusion or assertion? None provided. Suppose one were to create a SASA plot (as in Figure 3c) for the BTA-C6 in C5 system, will it look different than that for fiber (1)?

3)While the mechanism described in Figure 4d (caption) is believable (i.e., a fast 'adsorption' of the monomer on the surface & its lateral mobility on the oligomer, followed by insertion), the identification of 'hot spots' appears somewhat subjective.
Figure 3b or 3c are Delta E or SASA for fiber (1) obtained from CG method. Figure S2(a) or S2(b) are the same quantities for fiber (1) obtained by AA method. The nature of the graphs are quite different. There is only fluctuation in the AA data, while there appears to be several in the CG data (the oligomer sizes are different, but the point I am making holds good despite that). The magnitude of the fluctuation(s) as well as their frequency appear to be quite different between the AA and the CG approaches. Although a similar CG data for fiber (2) is provided in Figure S1(b) and (c), the corresponding AA data are not provided, but I would suspect that it would be similar to that in Figure S2(a) or S2(b). Thus, there is fundamentally different in the fluctuations between the AA and CG approaches, which has not been addressed in the manuscript.

4) Consider Figure 3d or S1d or S2c. These figures are used to identify molecules in the oligomer which can be characterized as 'hot spots'. In Figure 3d, the data is continuous. There is no discontinuity between the 'cold spots' and 'hot spots'. Thus, how are the energy or SASA cutoffs to determine hot spotness non-subjective?

5) Top few lines of Page 12 state that monomer insertion into cold spots is unfavourable and cites Figure S4a. However, the relationship between this assertion and the data shown in Figure S4a is unclear.

Reviewer #2 (Remarks to the Author):

In "Into the Dynamics of a Supramolecular Polymer at Submolecular Resolution”, the authors presented a computational study of monomer exchange in different variants of a synthetic supramolecular polymer. The authors used state-of-the-art modeling techniques to get insight into

the structure and dynamics of supramolecular assembly.
I believe the following major points should be addressed in order for this work to have an impact on the community and on the wider field and before I can recommend this paper for publication in Nature Communication. Without addressing these issues and in part extending the scope of this work, I believe this manuscript is more suitable for a more specialized journal.

1) The authors characterized the kinetics of two different polymers, labelled (1) and (2), and found that their results are consistent with previously published FRET mixing essay (Figure 1 cd). In order to validate their structural (and dynamical) models, I believe the comparison with all the experimental data presented in this paper should be more quantitative. I am not sure if the authors with their “infrequent metadynamics approach” can estimate the quantities reported, for example, in Figure 1cd. However, there are other techniques that can be used a posteriori, once the thermodynamics of the assembly process has been characterized by means of biased simulations, to recover kinetics information. One possibility is reported here:

Marinelli F, Pietrucci F, Laio A, Piana S (2009) A Kinetic Model of Trp-Cage Folding from Multiple Biased Molecular Dynamics Simulations. PLoS Comput Biol 5(8): e1000452. doi:10.1371/journal.pcbi.1000452

2) Accurate and useful structural models should be predictive. Which properties of these polymers (other than the kinetics discussed in the previous point) can be predicted from the proposed model and *quantitatively* related to existing experimental data?

3) To impact on the wider community, the authors should expand the Discussions part to address more general questions. Which general rules of supramolecular assembly can be learned from these specific systems? How these simulations and structural models can guide the design of better (in terms of reconfiguring their structures and self-healing) polymers? The authors should not only describe a rational way to improve the design of supramolecular polymers inspired by the understanding gained from the current simulations, but they should also propose specific variations of the polymers studied here to improve certain properties and carry out the necessary simulations to validate their predictions.

Reviewer #3 (Remarks to the Author):

Bochicchio, Salvalaglio and Pavan describe a detailed Molecular Dynamics study of monomer exchange in BTA fibers. This paper builds on previous work using atomistic simulations (Baker et al., Nat Comm 2015) and well-parametrized coarse-grain simulations (Bochicchio and Pavan, ACS Nano 2017). However, this paper addresses specifically the exchange mechanism within fibers. Because this is a rare event, the authors use well-tempered meta-dynamics.

The major claims and novelty of the manuscript are two-fold in my opinion:

1. Exchange of monomers (with solution or elsewhere in the fiber) in supramolecular polymers of BTA occurs via a “hot-spot” mechanism, where defects in the fibers are important for the rate of exchange; more 'perfect' fibers of (2) show much slower exchange. This mechanism has not been reported in such detail previously as far as I know. This is a thought-provoking finding, although the authors should briefly comment on the generality of this mechanism as there are also known examples of supramolecular block-cofibers where there is no exchange within the stacks; does this mean these fibers are more 'perfect'?

2. The use of WT-MetaD on supramolecular polymers is novel and I expect it to have a quick impact in this field as it can cover the strongly different time scales associated with the different stages of self-assembly. The authors describe the methods in detail, which appear appropriate to me and the limitations are clearly marked. They find semi-quantitative agreement with the

experimental data, and the timescales found emphasize how difficult it can be to observe spontaneous supramolecular nucleation/polymerization in water.

In my opinion, in spite of their recent other publications on this system using simulations, these findings warrant publication as a communication in Nat. Comm. subject to some minor revisions that could improve the manuscript.

1. As indicated above, please comment on the generality of the 'hot-spot' mechanism.

2. Of course, experimental evidence of the hot-spot mechanism would greatly improve confidence in the result. I suspect this is not a realistic demand, but could the authors suggest how the mechanism could be proven?

3. It is not clear how in the MetaD, a monomer is chosen to be 'activated'. Is this a random monomer from the fiber or a certain molecule on one of the 'hot-spots'. Please clarify.

4. A clear picture of the monomer at CG resolution would help to interpret the images of the suprapolymers. Could be shown as an additional panel or inset of Figure 3, for instance.

Textual issues:

1. I'm a bit confused by the term 'intrinsic dynamics' used throughout the manuscript. Do the authors mean dynamics intrinsic to this specific system only, or do they mean 'internal dynamics', i.e. dynamics of reshuffling monomers without a monomer leaving the assembly in general?

2. Page 2 “Point mutation” is a confusing term in the context and can be removed.

3. Page 9, From “Monomer mobility on the fibre surface is also extremely interesting”: Why are the data in Fig. 6 part of the discussion at the end? This seems more appropriate in the Results section.

4. The captions of the Figures are very long and contain methods, references and conclusions more often than not. As both the main text and the SI often repeat these methods and/or conclusions, one gets to read the same statements multiple times. Please trust the reader and use the caption more as a legend.

5. The last two sentences of the manuscript are stating the same. Also, I think the comparison with bone and skin tissue is very far-fetched, compared to an otherwise to-the-point manuscript.

Point-By-Point Response to the Referees' Comments

We carefully considered all Referees' comments and did our best to respond and satisfy in full their requests/concerns. Indeed, the Referees' comments helped us to make our paper better, and we would like to thank them for the useful suggestions.

All points raised by the Referees have been carefully considered and individually addressed as detailed below. Our comments are colored in blue, extracts from the text of the manuscript are *highlighted in yellow and italicized*. Our paper has been revised accordingly (changes in the text are highlighted in yellow in the marked revised manuscript enclosed to this resubmission).

Reviewer 1

This is an interesting manuscript which reports mechanistic, thermodynamic and dynamical details of monomer binding/unbinding transitions to a supramolecular oligomer. The work is carried out using MD simulations, and follows a previously published experimental work using the STORM technique. The simulations are carried out competently and the analyses are exquisitely done. Detailed insights are provided. The result shown in Figure 2 in particular is quite good & illuminating.

We are glad that the Referee finds our work interesting and well-conducted.

In its current form, the manuscript is not recommended for publication. However, in order to proceed further on a recommendation, some vital concerns need to be addressed. These are:

1) What is the purpose of reporting simulations of the BTA-C6 in C5? (Figure 2). How does it fit into the rest of the paper? This is unclear in the current MS.

To generalize our results, we studied supramolecular polymers with different-levels of structural complexity in different media. In particular, in Fig. 2a we show the mechanism of monomer exchange in the absence of any solvent (gas phase). From this "simple" supramolecular polymer model we clearly see that to exchange a monomer out from the fibre a defect must be first created along the stack. Thus, monomer exchange appears as a non-uniform process, but it is characterized by two distinct steps ((i) creation of the defect and (ii) jump out from the fibre).

The purpose of simulating this simple fibre as immerged in organic solvent (C_5) is to show that the mechanism of monomer exchange is the same also in organic solvent (in another intrinsically ordered condition). Namely, this demonstrates that, independently on the solvent, a defect is always necessary to exchange a monomer out from the fibre. This is interesting as this suggests the general character and importance of structural defects for monomer exchange.

We believe that these cases in organic solvent (C_5, at AA-level, and now we have added a case at CG-level in octane, C_8, to better compare with CG water-soluble fibres (**1**) and (**2**), see also below), while clearly different from more complex BTA fibres in water solution, are very important to provide generality to the concept. According to the Referee comment, we have better clarified the importance of these additional systems at the end of Page 3 and beginning of Page 4:

"We also studied the same system in organic solvent (pentane, C_5), where the BTA-C_6 monomers are soluble. In these conditions this system is known to form ordered/extended stacks.[9,25-26]... ...These two simulated systems are extremely useful to our study, as these allow investigating monomer exchange in intrinsically ordered, relatively simple supramolecular polymers (less structurally complex, for example, than water-soluble BTA fibres: see below)."

and at the end of Page 4 – beginning of Page 4:

"While here we are primarily interested in water-soluble supramolecular polymers, simulating these simpler systems is very useful to obtain a first molecular-level insight into the mechanism of monomer exchange in these supramolecular fibres, highlighting the importance of defects in the process."

2) Figure 2 are results from AA simulations in an organic solvent of a supramolecule which is largely hydrophobic, while the rest of the MS discusses hydrophilic polymers in water, modelled using coarse grained force field. On page 5 (one sentence prior to the section on "Intrinsic dynamics ..."), the authors state that water soluble fibers possess hot spots (there appears to be an allusion that the water soluble fibers have more hot spots than the C5 soluble ones, but the sentence is quite unclear). What is the evidence for this allusion or assertion? None provided. Suppose one were to create a SASA plot (as in Figure 3c) for the BTA-C6 in C5 system, will it look different than that for fiber (1)?

We thank the Referee for this observation. Clearly, this deserved more space and clarity, which has been now reserved in the revised version of our work. We also thank the Referee for the useful suggestion, which helped us improving our paper.

Based on the Referree's comments, we have performed additional analyses. First, we have repeated the analyses of Figure 3 for BTA fibre (**1**) on a shorter time period (200 ns of CG-MD). This is more correct to compare with fibre (**2**) (data collected from 2 µs of CG-MD), as the dynamics of fibre (**1**) is ~10 times faster than that of fibre (**2**). Then, inspired by the Referee's demand of objectivity in the identification of the hot spots (HS), we also calculated the core-core coordination of the individual monomers into the fibres (indicative of the order in the stacking of the individual monomers into the fibres). This parameter is easier to compare between the different fibres than ΔE (as the latter changes for different monomer structures). We put coordination in relation to the SASA for each monomer, the latter expressed in terms of average SASA (0 in the plots), and monomer deviation in % from the average (see new Fig. 3f and Fig. 5a). This analysis provides a clearer picture of stably-stacked monomers and of the monomers that unambigously correspond to hot-spots in the fibres (new Fig. 3f, in red: those with coordination ≤1 and SASA greater than the average), while this also allows to compare between different fibres (different size monomers).

According to the Referee comment, we performed the same analysis on the hot-spots (HS) also for the ordered BTA-C_6 polymer of Fig. 2. The AA models for the BTA-C_6 fibre in intrinsically ordered conditions (in the gas phase and organic solvent) did not show presence of any defect along the stacks (hot spot). However, for the most fair comparison, we also created a new CG model for BTA-C_6 which was simulated in organic solvent (C_8: a well-tested standard organic solvent in the MARTINI CG environment). In this way, we exclude any effect due to the AA *vs.* CG comparison (see also our response to the next point).

Comparing our CG models, the difference between CG BTA-C_6 and CG BTA water-soluble fibres is again striking. All CG BTA-C_6 monomers lay around the average SASA, ΔE and coordination values (see new Supplementary Fig. 3), while no HS are observed in these ordered oligomers (Fig. 3f: purple) – all monomers have coordination ~2. We thank again the Referee for this observation – we believe that thanks

to these additional analyses our work is now sensibly clearer. We have added the following sentence in the revised manuscript from Page 5, last 4 lines, to Page 6, first 5 lines:

*"It is interesting to relate the coordination number of each BTA core to the SASA (percentage deviation from the average) for the individual monomers in the fibre (**Figure 3f**). This allows to unambiguously identify defects in the fibre – monomers with coordination ≤1 and SASA greater than the average (**Figure 3f**: red) – and to compare between different fibres (different size monomers). For example, for a fair comparison with the ordered stacks of **Figure 2**, we also created a CG model for a BTA-C_6 fibre in organic solvent (see **Methods**). It is interesting to note that the same analyses from the CG-MD simulation of this system (**Supplementary Figure 3**) do not evidence any defects/breakages. Unlike fibre (**1**), CG fibre BTA-C_6 has all monomers coordination ~2 and SASA well consistent with the average value, which highlights the nearly perfect nature of these stacks (**Figure 3f**: purple)."*

3)While the mechanism described in Figure 4d (caption) is believable (i.e., a fast 'adsorption' of the monomer on the surface & its lateral mobility on the oligomer, followed by insertion), the identification of 'hot spots' appears somewhat subjective. Figure 3b or 3c are Delta E or SASA for fiber (1) obtained from CG method. Figure S2(a) or S2(b) are the same quantities for fiber (1) obtained by AA method. The nature of the graphs are quite different. There is only fluctuation in the AA data, while there appears to be several in the CG data (the oligomer sizes are different, but the point I am making holds good despite that). The magnitude of the fluctuation(s) as well as their frequency appear to be quite different between the AA and the CG approaches. Although a similar CG data for fiber (2) is provided in Figure S1(b) and (c), the corresponding AA data are not provided, but I would suspect that it would be similar to that in Figure S2(a) or S2(b). Thus, there is fundamentally different in the fluctuations between the AA and CG approaches, which has not been addressed in the manuscript.

We believe that now, with the new additional analyses mentioned above, and the comparison with the BTA-C_6 systems, the identification of the HSs in these water-soluble fibres is clearer and objective (new Fig. 3f).

The difference between AA and CG systems is not surprising and can be expected. In fact, the CG regime allows not only to simulate longer times, but the simplified representation of the molecules also improves the conformational sampling of the system. The result is that, in general, CG models are intrinsically more dynamic than AA ones. This is reflected by the larger oscillations into the systems and in the larger error bars in the data from the CG. As the Referee correctly noted, only a few "fluctuations" are present in the AA models. However, in the few hundreds of nanoseconds effectively accessible at this level of resolution, the AA system simply evolves toward the closest minimum energy configuration and vibrates around that state. On the other hand, in the CG systems we can see real dynamic motions and reconfigurations. With our CG models we can go from disassembled monomers to reliable equilibrated fibres easily (see *ACS Nano* **2017**, *11*, 1000). This is impossible using the AA models for these extremely complex systems, where we can only start from initially-extended perfect stacks that are further equilibrated via AA-MD. In general, one common risk of the limited sampling achievable via AA models (especially for very complex systems) is that these can remain "trapped" into local minimum energy configurations (even far from the global minimum energy one), appearing as more static than what they really are.

Anyways, the main goal in reporting the HS analysis on the AA model for fibre (**1**) (Supplementary Fig. 2) is that, despite their higher relative staticity, despite the fact that these start from a perfectly extended stacked configuration without any defect, despite the limited simulation times that can be achieved, these fibres immediately evolve during the AA-MD toward a condition where there are defects along the stack

(HSs). As in the CG models, these HSs are characterized by monomers having larger SASA and more weakly bounded to the rest of the fibre (more likely to exchange) compared to the average. This clearly demonstrates that in water these BTAs do not form perfectly extended stacks (this is a very unfavorable state for this system), but BTA polymers intrinsically possessing defects (HSs) in their structure.

We believe that this comparison between AA and CG is important to demonstrate that the natural formation of defects/HSs along these water-soluble fibres evidenced by our models is not somehow due to the CG models but is also present in the AA ones (even accounting for all sampling and timescales limitations of the latters).

In the revised text, we have added a clarification sentence on the meaning of this comparison at Page 5, lines 18-22:

*"Similar trend and defects in fibre (**1**) are evidenced also at AA-level (**Supplementary Figure 2**: AA-fibre (**1**)).[25] While AA models are clearly more static than CG ones (limited in terms of sampling, length and timescales that can be effectively explored), this demonstrates that even at such a high-resolution core stacking within BTA fibre (**1**) is far from being perfect in water."*

4) Consider Figure 3d or S1d or S2c. These figures are used to identify molecules in the oligomer which can be characterized as 'hot spots'. In Figure 3d, the data is continuous. There is no discontinuity between the 'cold spots' and 'hot spots'. Thus, how are the energy or SASA cutoffs to determine hot spotness non-subjective?

Our new analyses, comparing the monomer coordination and SASA (% deviation from the average) now eliminates any subjectivity in the presentation of the results and provide an objective determination of the HSs in these fibres (see above).

5) Top few lines of Page 12 state that monomer insertion into cold spots is unfavourable and cites Figure S4a. However, the relationship between this assertion and the data shown in Figure S4a is unclear.

We thank the Referee for this observation. To exchange one monomer from the internal core of the fibre, a new defect (HS) must be first created. Shown in new Supplementary Fig. 5a: this event has a characteristic timescale (t_0(**1**) or t_0(**2**) for the two fibres reported in Blue and Pink in the figure respectively) that is way longer than t_1 or t_1 (exchange of monomers on the surface). This event requires more energy than exchanging the monomers already present on the surface. We have clarified better this point at the bottom of Page 7, beginning of Page 8 by adding the following sentence:

*"…However, we could observe that Step 0 is very slow (t_0 ~0.1-1 s) compared to the latters (**Figure 5d**: t_1 and t_2), which makes exchanging monomers from the interior of the fibre (Steps 0-2) an unfavorable, less probable event than exchanging monomers already present on the fibre surface, either as adsorbed (Step 2) or stacked onto the hot spots (Steps 1-2) (see **Supplementary Figure 5a** for all computed timescales)."*

Reviewer 2

In "Into the Dynamics of a Supramolecular Polymer at Submolecular Resolution", the authors presented a computational study of monomer exchange in different variants of a synthetic supramolecular polymer. The authors used state-of-the-art modeling techniques to get insight into the structure and dynamics of supramolecular assembly.

I believe the following major points should be addressed in order for this work to have an impact on the community and on the wider field and before I can recommend this paper for publication in Nature Communication. Without addressing these issues and in part extending the scope of this work, I believe this manuscript is more suitable for a more specialized journal.

We thank the Referee for his/her constructive comments, which helped us to substantially improve our work and to extend its scope. To facilitate our response to the individual points raised by the Referee, we will start from point (2).

2) Accurate and useful structural models should be predictive. Which properties of these polymers (other than the kinetics discussed in the previous point) can be predicted from the proposed model and *quantitatively* related to existing experimental data?

It is fundamental to note that obtaining molecular-level information from the experimental characterization of soft polymeric assemblies in water (such as these fibres) is extremely difficult, if not impossible in most cases. These soft assemblies have "statistical" nature (fibres in solution have different lengths, are dynamic and differ one from the other). Thus, it is not possible to experimentally obtain meaningful molecular-level information for such systems, such as, for example, NMR or X-ray structures for proteins. In this sense, these fibres are more similar to amphiphilic assemblies (micelles, etc.) than to well-determined biomacromolecules.

From the structural point of view, all our AA and CG models for these BTA fibres have been already well-validated by comparison with all experimental evidence available for these systems, demonstrating very good agreement with the experiments (*e.g.*, SAXS profiles, fibre radii, helicity, persistence, etc. – see Ref. 25: *Nat. Commun.* **2015**, *6*, 6234; and Ref 31: *ACS Nano* **2017**, *11*, 1000). In particular, our models can be safely used to compare between different fibres or between the same fibre in different conditions, which is one of the best ways to validate the models while at the same time making predictions on the effect of the change in the system on the properties/behavior of the fibres. Our CG models for these fibres, consistent with the AA ones for all key factors controlling these supramolecular polymers (see Ref. 30: *JACS* **2016**, *138*, 13985), were proven to correctly capture the temperature sensitivity of the real fibres, and the effect of changing the monomer structure on the supramolecular polymer (Ref. 31: *ACS Nano* **2017**, *11*, 1000). For example, we found a remarkable linear trend between the local stacking persistency in our CG fibre models and the persistent lenghts of the real fibres as measured experimentally. This demonstrated that our models can be extremely useful to obtain trends to predict, for example, the persistent length of the fibres and the related mechanical properties as a function of structural modifications into the monomers.

In this perspective, before the submission of this work we already did everything that could be technically done to validate at best our AA and CG models from the structural point of view, while our models have been well-recognized as reliable. We have better clarified this in the revised version of our paper at page 5, lines 9-10, with the following sentence:

*"Recently, we developed all-atom (AA) and coarse-grained (CG) models of supramolecular polymers (**1**) and (**2**) consistent with the experimental evidences available for these systems (**Figure 3a**: GC monomer (**1**)).[25,30-31]"*

This concept is also recalled at page 8, lines 13-16:

*"We studied monomer exchange in supramolecular polymer (**2**), where (**2**) monomers differ from (**1**) only by a methyl group in the side chains (**Figure 1a**). Although at this level of accuracy this is a relatively subtle change, our CG models already demonstrated the ability to capture differences between fibres (**1**) and (**2**) consistent with the experimental trends.[31]"*

and in the Methods ad the end of page 14 lines 8-12:

"Based on the MARTINI force field,[49] these CG models also include the explicit treatment of inter-monomer H-bonding, and are consistent with the AA models[30] for all key factors controlling these supramolecular polymers (behavior of the monomers in solution, monomer-monomer interactions, cooperativity of H-bonding and self-assembly, amplification of order during supramolecular polymerization, etc.) and with the experimental trends.[31]"

1) The authors characterized the kinetics of two different polymers, labelled (1) and (2), and found that their results are consistent with previously published FRET mixing essay (Figure 1 cd). In order to validate their structural (and dynamical) models, I believe the comparison with all the experimental data presented in this paper should be more quantitative. I am not sure if the authors with their "infrequent metadynamics approach" can estimate the quantities reported, for example, in Figure 1cd. However, there are other techniques that can be used a posteriori, once the thermodynamics of the assembly process has been characterized by means of biased simulations, to recover kinetics information. One possibility is reported here:
Marinelli F, Pietrucci F, Laio A, Piana S (2009) A Kinetic Model of Trp-Cage Folding from Multiple Biased Molecular Dynamics Simulations. PLoS Comput Biol 5(8): e1000452. doi:10.1371/journal.pcbi.1000452

We thank the Referee for suggesting this relevant reference, which has been now properly cited (Ref. 41), however we would like to clarify a few important points related to the observations raised by the Referee.

The suggested paper describes an alternative method to perform a similar kinetic analysis to that we have already performed using infrequent WT-MetaD. Namely, this would provide exactly the same type of kinetic information (transition rates/timescales between states) that we are currently reporting in our work. In fact, our Figs. 4d and 5d show kinetic models for the monomer exchange process analogous to that for protein folding reported in Fig. 7 in the mentioned paper. Thus, in this case the suggested analysis would be redundant and would not provide an added value to our work. In particular, such analysis would not produce plots directly comparable to the fitting curves of the FRET experiments reported in our Fig. 1, but simply the transition rates for the exchange steps that we already have.

In light of the experimental limitations also mentioned above, it is fundamental to note that the semi-quantitative nature of our comparison with the experiments is not due to limitations in the modeling results nor to their accuracy, but rather to the semi-quantitative kinetic information that it is effectively possible to extract from the experiments. In particular, the multiexponential FRET fitting curves reported in Fig. 1 (taken from our previous work, Ref. 25: *Nat. Commun.* **2015**, *6*, 6234) are useful just to compare

between fibres (**1**) and (**2**). The unique information that can be safely extracted from these plots are that: (i) Monomer exchange is a complex process, likely involving multiple steps on different timescales (the nature of these multiple steps cannot be elucidated by the experiments) – this is consistent with the exchange steps seen in our models; (ii) While complex, the mechanism of monomer exchange seems to be very similar between fibres (**1**) and (**2**) (this is also correcly captured by our models, showing the same main exchange steps for both fibres); (iii) As all characteristic time constants in the multiexponential fitting curve of fibre (**2**) are roughly 1 order of magnitude slower than in fibre (**1**) (see Ref. 25: *Nat. Commun.* **2015**, *6*, 6234), this suggests that all key exchange steps are comparably slower in fibre (**2**) than in (**1**) (correctly captured by our models).

This has been now better clarified from Page 2, last line to Page 3, line 5:

*"The physical origin of this multi-exponential behaviour remained unclear. However, these experimental results suggested that: (i) Monomer exchange in these systems is likely not a single-step phenomenon, but rather a complex process involving multiple steps with faster/slower kinetics; (ii) Both fibres seem to exchange in similar fashion, while (iii) all main exchange steps are (comparably) slower in fibre (**2**) compared to fibre (**1**).[25]"*

Thus, it is clear that our models are fully consistent with all experimental observations that can be technically obtained for these fibres. Indeed, also from the kinetics point of view, we did everything possible to validate at best our models, well-demonstrating that these can be useful to compare between different fibres and to make predictions on, for example, the effect of structural modifications into the monomer on the dynamics of the supramolecular polymer. This is clarified in the text at Page 8, last 5 lines:

*"Shown in **Figures 5b-d**, all monomer exchange data collected for supramolecular polymer (**2**) reveal the same stepwise mechanism seen for fibre (**1**). However, in this case Steps 0-2 are all found ~1-2 orders of magnitude slower than in fibre (**1**) (**Figure 5d** and **Supplementary Figure 5**). This fits well with the difference of ~1 order of magnitude seen in all terms of the multi-exponential FRET exchange curves of the two fibres,[25] suggesting that the key exchange steps are all comparably slower in fibre (**2**) than in (**1**)."*

3) To impact on the wider community, the authors should expand the Discussions part to address more general questions. Which general rules of supramolecular assembly can be learned from these specific systems? How these simulations and structural models can guide the design of better (in terms of reconfiguring their structures and self-healing) polymers? The authors should not only describe a rational way to improve the design of supramolecular polymers inspired by the understanding gained from the current simulations, but they should also propose specific variations of the polymers studied here to improve certain properties and carry out the necessary simulations to validate their predictions.

We thank the Referee for the suggestion, in the revised version of our paper we did our best to generalize the results of our modeling. We believe that this made our work sensibly better, and we thank the Referee for his/her constructive suggestions.

Our models clearly show that some factors are key for the dynamics and exchange in these fibres (hydrophobic effects, H-bonding, level of order, etc.). While suggesting (and modeling) "real" alternative chemical monomer architectures in this paper without having the possibility to compare our results with the experiments would be unwise and far-fetched, we played with our molecular models, using them as toy-models, to understand what happens to the kinetics of exchange when specific types of interactions are

changed within the self-assembled fibres. The additional results we obtained are extremely interesting and sensibly improved the general message of our paper.

For example, we already know that augmenting the hydrophobicity of the side chains of the monomers slows down both the internal dynamics of the fibre and the exchange with the solvent (Figs. 3-5: fibre (**1**) *vs.* (**2**), where (**2**) monomers are more hydrophobic than (**1**)). Interestingly, in our revised paper we now describe a toy-model where we augmented the interaction between the cores into the fibre, without changing any other solute-solute or solute-solvent interaction (*i.e.*, preserving the hydrophobicity of the monomers). All this is well described in the **Results** in a dedicated subsection "*Key factors for supramolecular dynamics*" (Page 10) and in the **Discussion** (from the last 4 lines of Page 12 on).

We observed that Step 1 in the exchange – monomer escape from a hot-spot (HS) – was sensibly slowed down when the strength of the core-core interaction (ε: potential well depth for the interaction between the CG beads composing the monomer cores) was augmented (compared to all other interactions in the system), while Step 2 (the jumping in water of a monomer absorbed onto the fibre surface, and not stacked to any other core) was not affected at all (Fig. 7b). As monomer escape from the HSs becomes slower, the fibre surface also become more and more static (Figs. 7c,d) compared to the reference fibre (**1**). Augmenting further the core-core interaction (3ε) was found to make Step 1 even slower than Step 2 (see new Fig. 7b), changing the rate limiting step for the process of monomer exchange with water in this system (which now becomes t_1: the time necessary to have available "free" monomers absorbed on the surface to exchange with water). In these conditions, no shifting of monomers along the fibre is seen for the monomers of fibre 3ε (including those in the defects). The surface of the supramolecular polymer as described in Figs. 6 and 7 thus becomes completely static in the timescale of these CG-MD simulations.

On the opposite, weakening the core-core interaction (to 0.75ε) was found to accelerate monomer escape from the HSs. The fibre surface thus becomes more dynamic (see new Fig. 7b: dotted red, and Figs. 7c,d). The same effect is obtained when simply "turning off" the H-bonding between the cores in our toy-model (*i.e.*, by setting to 0 the partial charges into the dipoles mimicking H-bonding into the CG models of BTA (**1**), see Supplementary Fig. 7). All this is now extensively discussed in the **Results** and in the **Discussion**.

While clearly qualitative, the results obtained through this new toy model are extremely interesting. We believe that all this additional work now depicts a comprehensive scenario of the key factors controlling the dynamics of these fibre. These fibres are known to be able to dynamically rearrange their structure (monomer dynamic reconfiguration along the fibre) in response, for example, to the binding with a multivalent recruiter (*e.g.*, DNA, see: *PNAS* 2013, 110, 12203). Such dynamic properties are clearly controlled by the surface dynamics of these fibres. In this sense, we believe that our work provides an unprecedented, high-resolution description of what controls the fibre dynamics and useful cues to rationally design (or customize) the monomers to control such dynamic properties.

Using cutting-edge modeling and simulation techniques, we provide molecular-level description of the mechanism and kinetics of monomer exchange that has never been obtained before via any other method. We are consistent with all available experimental observations for these systems, and, for the first time, we show the monomer exchange process at nearly-atomic resolution "live" during the simulations (impossible with any experimental technique). We reveal general principles, (i) suggesting that in an assembly such as these supramolecular fibres the exchange starts from the defects present into the structure, (ii) showing the bioinspired nature of these fibres and now, thanks to additional work conducted inspired by the Referee's suggestions, (iii) also demonstrating/suggesting how to tune key interactions in the system to control the dynamics of these supramolecular polymers.

We believe that our revised work now fully accomplishes the requisite of generality and broad interest for the community of *Nature Communications*.

Reviewer 3

Bochicchio, Salvalaglio and Pavan describe a detailed Molecular Dynamics study of monomer exchange in BTA fibers. This paper builds on previous work using atomistic simulations (Baker et al., Nat Comm 2015) and well-parametrized coarse-grain simulations (Bochicchio and Pavan, ACS Nano 2017). However, this paper addresses specifically the exchange mechanism within fibers. Because this is a rare event, the authors use well-tempered meta-dynamics.

The major claims and novelty of the manuscript are two-fold in my opinion:

1. Exchange of monomers (with solution or elsewhere in the fiber) in supramolecular polymers of BTA occurs via a “hot-spot” mechanism, where defects in the fibers are important for the rate of exchange; more 'perfect' fibers of (2) show much slower exchange. This mechanism has not been reported in such detail previously as far as I know. This is a thought-provoking finding, although the authors should briefly comment on the generality of this mechanism as there are also known examples of supramolecular block-cofibers where there is no exchange within the stacks; does this mean these fibers are more 'perfect'?

We thank the Referee for the pertinent comment. Based on the results of our simulation, we can respond: Yes from a certain point of view, but not necessarily. Certainly, high perfection can be a reason for no-exchange in the fibre. However, no exchange can also be due to a too strong hydrophobic aggregation between the monomers, even if discontinuities/defects are present along the stack.

For example, comparing fibre (**1**) and (**2**) we observe that discontinuities/defects are present also in fibre (**2**), but here a little difference in terms of hydrophobicity of the monomers can produce a considerable reduced dynamics ((**2**) monomers are more hydorphobic than (**1**) – the HSs in fibre (**2**) are more static). Clear demonstration of the generality of this concept is also provided by our new toy models. Augmenting the relative strength of the core-core interaction (ε) between the monomers we obtain fibres that preserve the structural defects, but where the HSs become more and more static (see new Fig. 7: ε *vs.* 1.5ε,3ε).

In light of the additional HSs analyses conducted on water-soluble fibres and on BTA-C_6 in organic solvent (see also response to Reviewer 1), and of the results of our toy-models (see new Fig. 7, and related **Results** and **Discussion**), we can say that when there is exchange, this most likely originates from discontinuities/defects in the assembly (HSs), while reduced exchange may indicate higher structural perfection in the fibre (lower number of HSs), or that defects are present in the structure, but the monomers are too strongly incorporated into the assembly (*e.g.*, too strong hydrophobic effect – infrequent exchange) – see also below.

2. The use of WT-MetaD on supramolecular polymers is novel and I expect it to have a quick impact in this field as it can cover the strongly different time scales associated with the different stages of self-assembly. The authors describe the methods in detail, which appear appropriate to me and the limitations are clearly marked. They find semi-quantitative agreement with the experimental data, and the timescales found emphasize how difficult it can be to observe spontaneous supramolecular nucleation/polymerization in water.

In my opinion, in spite of their recent other publications on this system using simulations, these findings warrant publication as a communication in Nat. Comm. subject to some minor revisions that could improve the manuscript.

We thank the Reviewer for the very positive comments on our work, we are glad that the Reviewer finds this important, novel and interesting.

1. As indicated above, please comment on the generality of the 'hot-spot' mechanism.

We have now added some comments into the revised version of our paper on the generality of the hot-spots and their effect on the structure-dynamics – see Page 12, lines 10-14:

"Generalizing our findings, it seems reasonable to hypothesize that when exchange is observed in such supramolecular structures, this originates from defects (hot spots) that are dynamically formed/healed in the assembly. On the contrary, when no exchange is observed, this could be imputable to a too strong interaction between the monomers (e.g., hydrophobic, etc.), or high structural perfection within the assembly (lack of exchange hot spots)."

And at Page 11, lines 2-8:

"…These results well demonstrate that weakening/strengthening the interactions between the cores (compared to the other interactions in the system) makes the hot spots and, overall, the surface of these fibres more/less dynamic. … …This suggests that, in principle, by changing the monomer structure, and tuning the relative strength of the core-core interaction, it is possible to determine the surface dynamics in the fibres."

2. Of course, experimental evidence of the hot-spot mechanism would greatly improve confidence in the result. I suspect this is not a realistic demand, but could the authors suggest how the mechanism could be proven?

As the Referee correctly recognized, obtaining analogous submolecular-level evidence for the existence of the HSs into the structure of these fibres by the experiments is not realistic. However, indeed there are indirect experimental evidences supporting the existence of these exchange HSs in the real fibres. In fact, in the recent STORM work by Albertazzi *et al.* (Ref. 22: *Science* **2014**, *344*, 491), the authors hypothesized (without having the possibility to prove this) that the stacking along these fibers has to be dynamic and non-perfect, to some extent, in order to explain the uniform exchange pathway seen all along these BTA fibres (fibre (**1**)). Namely, if the fibres exchange uniformly all along their length, there must be in/out gateways to exchange along the fibre: the same HSs now clearly identified in our models. We now better stressed this important point in the revised verion of our work at Page 8, lines 7-11.

*"These findings indicate that monomer exchange preferentially originates from the numerous hot spots identified in our models (defects) present all along the fibre surface. By bridging the gap between structure and dynamics, our approach provides molecular-level insight consistent with the uniform exchange along fibre (**1**) seen by STORM, explicable only under the conditions that core stacking is not perfect and defected points prone to monomer exchange (our "hot spots") are present all along these supramolecular polymers.[22]"*

3. It is not clear how in the MetaD, a monomer is chosen to be 'activated'. Is this a random monomer from the fiber or a certain molecule on one of the 'hot-spots'. Please clarify.

In the biased fibre-to-water exchange, as the activated monomer we chose a monomer onto a HS (an average representative one in the red/blue ensamble of Figs. 3 and 5a). This has been now better clarified in the text at Page 6, line 13-16, where we added the following sentence:

*"Starting from system (**1**), we chose one monomer (**Figure 4d**: green) corresponding to a surface hot spot in equilibrated CG-fibre (**1**) (**Figure 3**: red). WT-MetaD simulations activating monomer exchange from the hot spot revealed that monomer exchange with water is not a single step process (**A-C**)."*

On the other hand, for the reincorporation, the monitored monomer is simply a dessolved monomer which is left to incorporate onto the fibre.

4. A clear picture of the monomer at CG resolution would help to interpret the images of the suprapolymers. Could be shown as an additional panel or inset of Figure 3, for instance.

This has now been added in Fig. 3a.

Textual issues:

1. I'm a bit confused by the term 'intrinsic dynamics' used throughout the manuscript. Do the authors mean dynamics intrinsic to this specific system only, or do they mean 'internal dynamics', i.e. dynamics of reshuffling monomers without a monomer leaving the assembly in general?

We agree with the Referee that the term "intrinsic" can be confusing. Indeed, with "intrinsic" we meant the internal dynamics (monomer reshuffling) of the fibre. Everywhere in the text now we replaced for clarity *"intrinsic"* with *"internal"* and we clearly specified it when talking about exchange with water (exchange in and out the fibre).

2. Page 2 "Point mutation" is a confusing term in the context and can be removed.

This has been removed.

3. Page 9, From "Monomer mobility on the fibre surface is also extremely interesting": Why are the data in Fig. 6 part of the discussion at the end? This seems more appropriate in the Results section.

This part has been entirely moved in the **Results** in a dedicated section entitled "*Internal dynamics of water-soluble BTA supramolecular polymers*".
".

4. The captions of the Figures are very long and contain methods, references and conclusions more often than not. As both the main text and the SI often repeat these methods and/or conclusions, one gets to read the same statements multiple times. Please trust the reader and use the caption more as a legend.

We agree with the Reviewer's suggestion. We did our best to shorten the figure captions while preserving all key information to allow the reader to understand the meaning of the figure panels.

5. The last two sentences of the manuscript are stating the same. Also, I think the comparison with bone and skin tissue is very far-fetched, compared to an otherwise to-the-point manuscript.

We agree with the Reviewer. The text reorganization in the revised version of our manuscript solved this issue. Also, we have also removed that far-fetched sentence.

REVIEWERS' COMMENTS:

Reviewer #1 (Remarks to the Author):

The revised manuscript clarifies many of the concerns expressed earlier. In particular, the difference in 'hotspots' between BTA in organic solvent and in water is quite evident from the results presented in the revised MS. The manuscript can be published now.

However, two minor concerns can be addressed in a revision (I do not need to see the revisions).

1) Page 6, Paragraph before section titled "Monomer exchange in a water-soluble BTA supra fibre": Sentence says that "creation of defects" are required gas phase/organic-solvent. Earlier, it was mentioned that such a system "do not show evidence for any defects/breakages". If so, how does monomer exchange occur in such systems? Sentence must be clear.

2) In the Discussions, physical insights into the difference between monomer exchange process in organic solvent and in water must be offered. Is it due to shorter relaxation times in water (lower viscosity). Is the hot spot phenomenon a general one which can be observed in systems where solvent relaxation times are comparable to supramolecule shape fluctuation times?

Reviewer #2 (Remarks to the Author):

While in principle I disagree with authors on the fact that the kinetics approach I proposed would provide the same kinetics information of infrequent WT-MetaD, I understand their concerns about extracting quantitative information from the reported experiments. So, I will not insist on this front. On the other side I am fully satisfied with their (structural) validation of the model, reported in previous works and now better clarified in the revised manuscript. I am also extremely pleased by the effort of the authors in addressing the problem of extending their analysis to capture general rules of supramolecular assembly that could guide the design of novel polymers with specific features. Therefore I recommend publication of the manuscript in the current form.

Reviewer #3 (Remarks to the Author):

All the points I raised were addressed and the clarity of the manuscript has been improved by the textual changes. The addition of the toy model is very valuable in creating not only a qualitative, but also to some extent a quantitative picture of how to control the dynamics of supramolecular polymers. It is an approach that quickly leads to more insights into any system and should be used more often by others. It has therefore raised the impact of the manuscript and I would recommend publication of the current manuscript.

Point-By-Point Response to the Referees' Comments

All comments by the Referees and the Editor have been carefully considered and individually addressed as detailed below. Again, we would like to thank the Referee and the editorial office for the useful suggestions, which helped us to improve further our work. Our comments are colored in blue, extracts from the text of the manuscript are *highlighted in yellow and italicized*. Our paper has been revised accordingly (changes in the text are highlighted in yellow in the marked revised manuscript enclosed to this resubmission).

Reviewer #1 (Remarks to the Author):

The revised manuscript clarifies many of the concerns expressed earlier. In particular, the difference in 'hotspots' between BTA in organic solvent and in water is quite evident from the results presented in the revised MS. The manuscript can be published now.

We thank the Referee for the positive comments and for recommending publication of our work.

However, two minor concerns can be addressed in a revision (I do not need to see the revisions).

1) Page 6, Paragraph before section titled "Monomer exchange in a water-soluble BTA supra fibre": Sentence says that "creation of defects" are required gas phase/organic-solvent. Earlier, it was mentioned that such a system "do not show evidence for any defects/breakages". If so, how does monomer exchange occur in such systems? Sentence must be clear.

We thank the Referee for pointing out this unclear point. In our MetaD simulations we clearly observe that the formation of a (metastable) defect along the stack is a first necessary step to the exchange of monomers with the solution along the fibre. However, in our unbiased simulations in organic solvent (both AA-MD and CG-MD) we do not observe the spontaneous formation of persistent defects in BTA-C_6 stacks within the timescales accessible with our model (up to tens of µs). This means that these fibres do not intrinsically possess persistent stacking defects along their length contrary to water-soluble BTA fibres. The creation of defects along these ordered BTA-C_6 stacks is a transient/dynamic event that, while possible, pertains to longer time regimes than those accessible with our CG-MD. As indicated by the Referee, we modified two sentences in the first part of Page 6 to clarify this point.

*"It is interesting to note that the same analyses from the CG-MD simulation of this system (**Supplementary Figure 3**) do not evidence the spontaneous formation of any persistent defect/breakage in the timescales accessible with our model (microseconds)."*

"Thus, while in intrinsically ordered fibres (BTA-C_6 in gas phase and organic solvents) we observe that monomer exchange can proceed provided that a defect is first created along the stack, water-soluble BTA fibres intrinsically possess a number of hot spots where monomer exchange is more likely to occur."

2) In the Discussions, physical insights into the difference between monomer exchange process in organic solvent and in water must be offered. Is it due to shorter relaxation times in water (lower viscosity). Is the hot spot phenomenon a general one which can be observed in systems where solvent relaxation times are comparable to supramolecule shape fluctuation times?

This is an interesting question. Although the different solvent relaxation times may certainly play a role in the exchange process, based on our results it seems clear that many factors (and combination thereof), rather than a single one, indeed contribute in controlling the exchange process. Thanks to our models, we observe that the presence of hot spots along BTA supramolecular polymers in water is due to a complex interplay between various key factors – *e.g.*, the length and flexibility of the water-soluble BTA side chains, directional vs. non-directional forces in the interaction between the monomers and monomer interactions with the solvent (hydrophobic effect), that competes with the directional interactions between the BTAs leading to fibre folding.

We agree with the Referee that adding these observations can be important to the readers. Thus, as suggested by the Referee, we added a sentence on this point in the Discussion section at page 12:

"While in these intrinsically ordered conditions the dynamic creation of defects is key to the exchange process, in water BTA supramolecular polymers intrinsically possess a certain number of discontinuities. These are due to the complex interplay of different factors, such as the length/flexibility of the BTA side chains, the competition between directional and non-directional interactions between the BTAs, and their interaction with the solvent (hydrophobicity). These structural defects work as hot spots for monomer exchange (Figure 3)."
